# Study protocol of a cluster randomized controlled trial to evaluate effectiveness of a system for maintaining high-quality early essential newborn care in Lao PDR

**DOI:** 10.1186/s12913-018-3311-7

**Published:** 2018-06-25

**Authors:** Sayaka Horiuchi, Sommana Rattana, Bounnack Saysanasongkham, Outhevanh Kounnavongsa, Shogo Kubota, Julie Cayrol, Kenzo Takahashi, Mariko Inoue, Asuka Nemoto, Kazue Yamaoka

**Affiliations:** 1 Teikyo University Graduate School of Public Health, 2-11-1 Kaga, Itabashi, Tokyo, Japan; 2 Department of Health Care, Ministry of Health, Ban thatkhao, Sisattanack District, Rue Simeuang, Vientiane, Lao PDR; 3 Maternal and Child Health unit, World Health Organization Country Office for Lao PDR, Ban Phonxay, That Luang Road, Vientiane, Lao PDR; 4 Center for International Child Health, Department of Paediatrics, The University of Melbourne, 50 Flemington Road, Parkville, VIC 3052 Australia

**Keywords:** Newborn care, Continuous quality improvement, Hospital management

## Abstract

**Background:**

Reduction in neonatal deaths has been a major challenge globally. To prevent neonatal deaths, improvements in newborn care have been promoted worldwide. The World Health Organization Western Pacific Regional Office has been promoting the Early Essential Newborn Care (EENC), a package of specific simple and cost-effective interventions, in their region. However, mere introduction of EENC cannot reduce neonatal deaths unless quality of care is ensured. In Lao PDR, the government introduced self-managed continuous monitoring as a sustainable way to improve the quality of care described in the EENC.

**Methods:**

A clustered randomized controlled trial was designed to compare the effectiveness of self-managed continuous monitoring with external supervisory visits to monitor health workers’ satisfactory EENC performance and their knowledge and skills related to the EENC in Lao PDR. Determinants of EENC performance will be measured with a structured questionnaire developed based on the Theory of Planned Behaviour, which predicts future behaviour. During self-managed continuous monitoring activities, health workers in each district hospital will conduct periodical peer reviews and feedback sessions.

Fifteen district hospitals will be randomly allocated into the self-managed continuous monitoring (intervention) and the supervision (control) groups. Fifteen health workers routinely involved in maternity and newborn care including physicians, midwives and other health staff will be recruited from each hospital (effect size 0.6, intra-cluster correlation coefficient 0.06, 5% alpha error and 80% power). We will compare the change in the mean score of the determinants before and one year after randomisation between the two groups. We will also compare the retention of knowledge and skills related to the EENC between the two groups. The expected enrolment period is July 20th, 2017 to July 20th, 2018.

**Discussion:**

This is the first cluster randomized trial to evaluate a self-managed continuous monitoring system for quality maintenance of newborn care in a resource-limited country. This research is conducted in collaboration with the Ministry of Health and international organizations; therefore, if effective, this intervention would be applied in larger areas of the country and the region.

**Trial registration:**

This trial was registered at UMIN-CTR on 15th of June, 2017. Registration number is UMIN000027794.

**Electronic supplementary material:**

The online version of this article (10.1186/s12913-018-3311-7) contains supplementary material, which is available to authorized users.

## Background

While the mortality rate for children under the age of five years has almost halved in the last 20 years globally, the neonatal mortality rate has not had such a dramatic reduction. In fact, the proportion of under-five child deaths due to neonatal mortality has increased worldwide [1, 2]. Approximately 7000 neonates died per day in 2015 mainly due to prematurity, birth-related complications (birth asphyxia) and neonatal sepsis [2]. In the Western Pacific Region, more than 70% of child deaths are due to neonatal complications, and one neonate dies every two minutes [3, 4]. Given that complications in the first month of life have become the leading cause of death in children under five, actions to improve neonatal health have become tremendously important to achieve Sustainable Development Goal 3 in 2030 [5]. Simple methods can be applied to prevent neonatal deaths and complications due to preventable factors within the first 24 h of birth, during which about half of all neonatal deaths occur [3, 6, 7].

Essential Newborn Care (ENC) is an evidence-based and effective measure to prevent newborn deaths and is recommended globally [8–11]. Helping Baby Breathe and the Neonatal Resuscitation Program are wide-spread educational programs that provide training materials for health care providers that focus on improvements in neonatal resuscitation in addition to providing information on essential skills [8]. Early Essential Newborn Care (EENC) aims to prevent early neonatal deaths by changing routinely implemented harmful practices through coaching involving a set of specific simple actions in birth assistance. The EENC does not require special medical equipment or medications and can be implemented in resource-limited countries. It is an officially recognised program by the World Health Organization (WHO) and the United Nations Children’s Fund (UNICEF) and is distinguished from ENC. The scale up of EENC activities is led by the Western Pacific Regional Office (WPRO) of the WHO, and 27,000 health workers in over 2000 facilities have been trained under this program in 8 prioritised countries in the region [9, 10]. It is estimated that at least 50,000 neonatal deaths could be averted by applying the EENC each year in the Western Pacific Region [9].

Lao People’s Democratic Republic (Lao PDR) had the highest neonatal mortality rate (27.2 neonatal deaths per 1000 live births in 2012) in the WHO Western Pacific Region [3, 9]. In 2015, adopting the EENC in Lao PDR to accelerate the reduction in neonatal deaths was therefore seen as a priority. The country is divided into 18 provinces, which are themselves divided into smaller districts. The Lao PDR health facilities are composed of central hospitals in the Vientiane capital, provincial hospitals in each province capital and district hospitals. The EENC was introduced in central hospitals and then expanded to 17 of the 18 provinces in 2016. The government committed to a plan of rolling out the EENC to district hospitals in 2017.

Along with the expansion of the program, retention of knowledge and practical performance have become issues of interest to provide quality in EENC, especially at the district level. Previous studies showed that neonatal resuscitation training improved participants’ knowledge, skills and performance; however the long-term effect of such training on the retention of knowledge, skills and performance remains uncertain [11–13]. Another study suggested that training improved the level of knowledge and skills but did not change daily practice [14]. These studies suggested that different mechanisms to maintain skills, knowledge and performance of EENC for a prolonged period after training would be necessary. The introduction of EENC can only be effective in reducing neonatal deaths long-term if routine EENC of high quality is maintained. Health workers’ competency is known to be critical for service quality [15]. Core competencies for midwifery care have been defined by the International Confederation of Midwifes as the demonstration by health workers of knowledge, skills and appropriate behaviour [16].

Although an external supervisory visit is a common method to promote behavioural changes among health workers and to maintain quality of care in many countries, it is not feasible in resource-limited settings due to budgetary and human resource demands. Furthermore, systematic reviews examining the use of supervision as a method to improve quality of care in low- and middle-income countries reported mixed evidence of the effect of supervision on quality improvement [17–19]. In Lao PDR, there is only a limited capacity to implement routine supervision. In addition, efforts made by district hospitals to solve problems after the supervisory visits are insufficient and, eventually, problems remain unsolved [20].

Self-managed continuous monitoring requires a lower budget and is considered more feasible than external supervisory visits. To build a sustainable system for continuous quality improvement, self-managed continuous monitoring was introduced in Lao PDR with support from the WHO. Self-managed continuous monitoring is expected to improve health workers’ knowledge and technical skills and to promote daily EENC practice [21]. However, little is known about the effectiveness of self-managed continuous monitoring on quality of care compared to that of external supervisory visits.

### Study objectives

This study aims to test whether in Lao PDR, a resource-limited country, self-managed continuous monitoring of activities for the EENC conducted by health workers involved in maternity and newborn care in district hospitals improves health workers’ behaviour (routine performance of standardised EENC) as well as retention of knowledge and skills after the EENC coaching. We have set determinants of EENC performance as a primary outcome because previous studies have pointed out that changing behaviour of health workers is difficult but key to providing appropriate care for patients. The determinants are factors that can predict future performance of EENC. We have selected Lao PDR because it has been a prioritised country in the Western Pacific Region and EENC has been strongly promoted in that country.

The specific objectives of this cluster randomized trial are:

1. To test whether self-managed continuous monitoring is effective in changing determinants of EENC performance amongst health workers at the district level compared with supervision.
2. To test whether self-managed continuous monitoring is effective in improving retention of technical knowledge and skills for EENC amongst health workers at the district level compared with supervision.
3. To specify the EENC components for which self-managed continuous monitoring is most effective for retaining knowledge and skills.
4. To evaluate whether self-managed continuous monitoring improves the environment required to enable EENC implementation in district hospitals. An enabling environment is one with policy commitment, availability of commodities and equipment and human resources in district hospitals.

We expect that our findings will inform decision makers on the effectiveness of the self-managed continuous monitoring model at the district level and provide them with useful information to then design an effective monitoring system. We trust the lessons learnt from Lao PDR to be applicable to other resource-limited countries.

## Methods/design

### Trial design

This study is a cluster randomized controlled trial with four strata based on province and history of attendance for EENC coaching. It aims to evaluate superiority of self-managed continuous monitoring over supervision. It is a two-armed parallel group study. Allocation ratio is 1:1 within the same stratum.

### Study setting

This study targets district hospitals in Xiengkhuang and Huaphanh provinces of Lao PDR. The level of maternal and child health coverage of these two provinces corresponds to the national average [22], and the government usually selects these two provinces to test new interventions.

The clusters, or district hospitals, will be divided into four strata by location (Xiengkhuang or Huaphanh) and by history of attendance of EENC coaching. Clusters will then be chosen from each of the strata. We will evaluate the overall effects of the intervention on the outcome but will not conduct stratified analyses.

Of note, the two provinces are different in several aspects such as road conditions, culture and economic status. Huaphanh province has a larger number of poor and remote villages, and health outcomes in this province are worse than in Xiengkhuang [22, 23]. Indeed, capacity of health staff is said to be higher and medical facilities better equipped in Xiengkhuang [24, 25]. To remove the effect of these differences between the two provinces, the clusters are divided by province.

Two district hospitals in each province received EENC coaching in 2016. The other district hospitals will receive EENC coaching upon entry into this study. Within one and a half days, the EENC coaching provides information on the theoretical rationale for EENC and teaches health workers EENC technical skills. Given that district hospitals that have already received EENC coaching might have different levels of determinants of EENC performance, knowledge and skills compared to districts that have not, the district hospitals will be divided based on a history of attendance to receive EENC coaching.

This study is led by the Teikyo University Graduate School of Public health in collaboration with the Lao Ministry of Health (MoH) and the WHO. The MoH has reviewed and agreed on the study protocol. Although the research team will mainly lead this study, the MoH will be responsible for conducting the EENC coaching and supervision in district hospitals. MoH will also be involved in incorporating the study results into the country’s policy.

### Participants and eligibility criteria

#### Inclusion criteria

Health workers who are routinely involved in maternal and newborn care and have received EENC coaching in the target district hospitals are eligible to join this study. Health workers include obstetricians, paediatricians, midwives, nurses and other types of health workers. The eligible participants need to provide written informed consent.

#### Exclusion criteria

To minimise loss to follow up, we will exclude those who are scheduled to leave their current workplace or retire within one year.

### Recruitment and allocation

#### Recruitment

After identifying eligible participants, written informed consent will be obtained from each. To obtain sufficient numbers of participants from each district hospital and to minimise loss to follow up, we will involve all district hospital directors. Directors will usually monitor, supervise and encourage their staff in their duties. Getting directors’ participation is therefore critical. Researchers, together with provincial and district staff, will set up a schedule to ensure that all participants are in the district hospital at the time of data collection or supervision. In the intervention group, central and provincial staff will also remind district directors and local staff to conduct self-managed continuous monitoring and submit reports. The expected enrolment period is July 20th, 2017 to July 20th, 2018.

#### Allocation

**Fig. 1:**
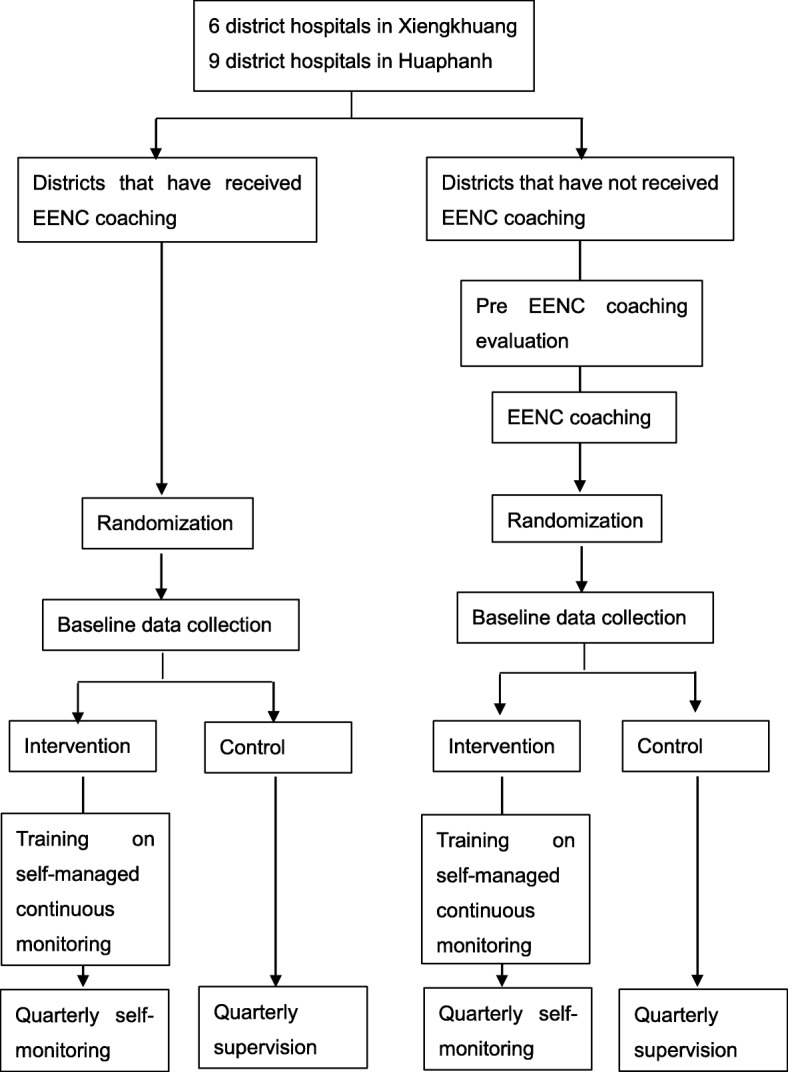
Trial flow

There will be 6 clusters (district hospitals) in Xiengkhuang and 9 clusters in Huaphanh province. Clusters in each province will be divided into two strata based on history of attendance of EENC coaching, and then will be randomized to two intervention groups (self-managed continuous monitoring and supervisory visits) within each stratum following random allocation rule with urn model. Figure 1 summarizes how district hospitals will be allocated to two intervention groups.

#### Blinding

Blinding of participants would be difficult in this study due to the nature of the intervention. We will choose assessors amongst central and provincial officials who do not know about the allocation. Standardised checklists will be used for outcome measurement to minimise bias. Team members who manage data will be blinded until the code is open. Members who analyse data will also be kept blinded.

#### Intervention

##### Intervention group: Self-managed continuous monitoring

Self-managed continuous monitoring is monitoring conducted by the recruited health workers in each district hospital following the Ministry of Health guidelines (Additional file 1). In the self-managed continuous monitoring group, district health workers will form a multi-disciplinary team with representatives from both maternity and paediatrics and the team will hold quarterly feedback meetings to discuss solutions and make action plans based on the results of the periodic peer evaluation of clinical practice and interviews of mothers of neonates. After randomisation, training to introduce self-managed continuous monitoring will be conducted for the intervention group. The peer evaluation and interviews will be conducted at every birth after randomisation. Provincial officials will remind the districts to submit quarterly reports after their self-managed continuous monitoring activities.

##### Control group: Supervisory visit

After randomisation, supervision will be provided every three months to the control group. Members of the Provincial Health Department and/or the Provincial Hospital will visit district hospitals to observe whether district health workers are providing appropriate care through direct observation and/or chart review and whether there are sufficient essential medicines and equipment available using standardised checklists. Provincial supervisors will also interview mothers who gave birth at a hospital to determine whether they have received appropriate care. Provincial supervisors will give feedback to district health workers as needed and ask them to solve any problems that are detected.

### Endpoints

The primary endpoint is the change in determinants of EENC performance amongst health workers. Secondary endpoints are 1) EENC knowledge, 2) EENC skills and 3) hospital environmental changes facilitating implementation of EENC. We will also compare the cost of monitoring activities between the two groups. Measures and schedule of data collection are explained in the next section.

### Participant timeline

**Fig. 2:**
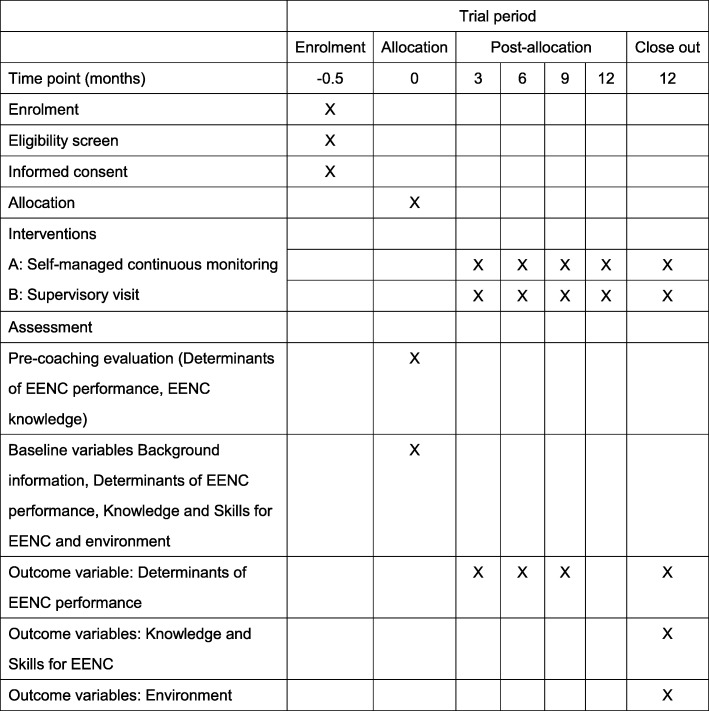
Trial schedule

Figure 2 shows the trial schedule. After identifying potentially eligible participants, a trained research staff member at the central or provincial level will explain the goals and procedure of the study to them, invite them to participate and obtain written consent from those who desire to participate after the explanation.

District hospitals that have not yet received EENC coaching will receive coaching before randomisation. Pre-coaching evaluation will be done to measure determinants of EENC performance and knowledge related to neonatal care before the introduction of EENC.

After the coaching, the districts will be randomly allocated into two groups. A research team member will distribute and explain a structured questionnaire to be filled out by the district participants to measure determinants of EENC performance and will administer written and simulation tests to measure EENC knowledge and skills. The collected data will be used as baseline data.

District hospitals that are allocated into the intervention group will receive a half day of on-the-job training on self-managed continuous monitoring. A research staff member will explain the concept, schedule, procedure and checklists of self-managed continuous monitoring.

After randomisation, district hospitals in the intervention group will conduct self-managed continuous monitoring and submit quarterly reports to the province. In the control group, provincial supervisors who are not involved in the research will visit districts every three months to supervise implementation of EENC and give feedback to district health workers.

Study participants will be monitored until one year after entry into this study or withdrawal from the study for any reason. The date of the final evaluation will be determined once a schedule for enrolment is set for each district. We will allow a variation of a month around the endpoint for timing of the final evaluation. The outcome data on participants who withdraw from the study before one year will be collected at the time of withdrawal.

### Sample size and power calculation

The number of clusters, or district hospitals, is 15. We will recruit 15 health workers from each of the 15 district hospitals, 225 in total. This study is designed to detect at least a 5-point increase in the score for determinants of EENC performance (behaviour change) in the intervention group compared to the control group with 80% statistical power at a significance level of 0.05 using a 2-tailed test. There has been no previous cluster randomized studies carried out in Lao PDR. Therefore, we assume that the intra-cluster correlation coefficient (ICC) is 0.06 and that the effect size is 0.6 by referring to previous cluster RCTs in developing countries [26]. We examined the statistical power of this cluster size with this ICC and effect size. In previous studies that used the questionnaire of Planned Theory Behaviour, the largest variance in the score was 6.86 [27]. Given that variance, we expect to detect a difference of 4.1 points in the score for determinants of EENC performance with an effect size of 0.6, which is smaller than the 5-point difference we expect to detect**.** We, therefore, believe that this sample size is large enough to test the primary outcome.

### Data collection, management and analysis

#### Data collection

Data will be collected from results of a questionnaire, written and simulation tests and checklists by central and provincial officials, who will have been trained prior to commencing the study. An in-depth interview of health workers is planned to take place at the final evaluation to identify key factors in the success of implementing activities related to the EENC.

##### Determinants of EENC performance amongst health workers

Determinants of EENC performance by health workers will be quantified by using a structured questionnaire (Additional file 2) that was developed based on the Theory of Planned Behaviour. [27–30] The questionnaire will measure determinants of behaviour: attitude, subjective norms, perceived behaviour controls and behaviour intentions that would influence future behaviour. The higher the total score of the determinants, the higher the likelihood of staff practising EENC in their daily work. Data will be collected six times from both groups during the study period: 1) before EENC coaching, 2) immediately after randomisation, 3) three months after randomisation, 4) six months after randomisation, 5) nine months after randomisation, and 6) one year after randomisation. Provincial officials will remind by telephone each district to fill out their own questionnaire every three months. Administered questionnaires will be saved in each district until provincial supervisors or assessors visit the district, at which point they will collect the questionnaires and submit them to the investigators. In the intervention group, checklists used for the self-managed continuous monitoring and interviews with pregnant women who receive the EENC will be stored and compared with the results of the questionnaire.

##### Knowledge of EENC amongst health workers

Data will be collected three times in both groups during the study period: 1) before EENC coaching, 2) immediately after randomisation, and 3) one year after randomisation. We will use written tests to quantify the level of knowledge of EENC (Additional file 3). The data will be collected when provincial officials visit the districts.

##### Skills for EENC amongst health workers

Data will be collected twice in both groups during the study period: 1) immediately after randomisation and 2) one year after randomisation. We will use simulation tests to quantify the level of skills for EENC (Additional file 4). The data will be collected when provincial officials visit the districts.

##### Hospital environmental changes facilitating implementation of EENC

The data will be collected twice in both groups during the study period: 1) before randomisation and 2) one year after randomisation by the provincial officials. We will use a checklist designed by the Lao MoH and the WHO to quantify the district hospital’s level of commitment and the availability of essential equipment, commodities, medications and human resources in each facility (Additional file 5).

We will also compare the cost spent for maintenance activities between the intervention and control groups.

##### Potential confounders

We will collect participants’ demographic data such as age, sex, ethnicity, title, position and professional experience at baseline. A self-administered questionnaire will be used (Additional file 6). Data on those who discontinue participation will be collected at the time of dropping out.

#### Data management

Collected paper data will be handed to the collaborating organisation and stored in the WHO Lao County Office. All documents will then be moved to the office at Teikyo University Graduate School of Public Health for data entry and coding. Double data entry will be conducted to minimise error. Personal information will be anonymised by allocating an identification number for each participant. A list of identification numbers is available when a participant must be identified. We will keep both paper and electronic data in the research office of the Teikyo University Graduate School of Public Health in a secure manner. Data will be kept at least five years after study completion, then discarded. Anonymised data could be shared with collaborating organizations such as the Lao MoH and the WHO.

#### Data analysis

To ensure that cluster randomisation is successful, the significance of differences between the intervention and control groups for the baseline characteristics of participants such as age, sex, position, experience in years will be examined using the chi-squared test and *t*-test.

The primary endpoint is the change in determinants of EENC performance (behaviour change) from baseline to one year after randomisation. We expect a larger increase in the score of determinants of EENC performance in the intervention group than in the control group. The difference between the two groups will be examined based on an intention to treat (ITT) principle with the full analysis set. The imputation of missing data will be performed using the last observation carried forward method (ITT/LOCF) and a multiple imputation method (ITT/MI) using chained equations under the assumption of missing at random.

We will use a linear random-effects mixed model employing the maximum likelihood method. We will perform a crude analysis and then adjust the results with baseline variables and other multiple variables. The same method will be used for analysing secondary outcomes such as changes in scores for skills and knowledge of EENC. Changes in the average score of the written and simulation tests between baseline and one year after randomisation will be compared between the intervention and control groups.

Sensitivity analyses will be planned using the per protocol set (PPS). A significance level of 5% with two tailed testing will be applied for all significance analyses. We will also perform subgroup analyses to assess whether the effect of self-managed continuous monitoring on EENC quality varies amongst the five main components of EENC (clean birth process, provision of warmth, support for early initiation and exclusiveness of breastfeeding, hygiene cord care and basic neonatal resuscitation).

All statistical analyses will be performed using SAS version 9.4 for Windows (SAS Institute, Cary, NC, USA).

#### Confidentiality

Personal data will be anonymised to ensure participants’ confidentiality. The list of identification numbers will be created electronically and saved separately from the rest of the data. Only anonymised data will be used for dissemination of outcomes. The list of identification numbers will not be shared with any stakeholders and will be discarded five years after completion of this study.

### Monitoring

#### Data and safety monitoring

The monitoring team is independent from the investigators and coordinating organizations. The monitoring team consists of three members from the Lao PDR MoH, The Center for International Child Health (The University of Melbourne) and Teikyo University. They will monitor the number of neonatal deaths in the target districts every three months after the start of the study. Should a significant difference in neonatal mortality arise between before and after this study or between the intervention and control groups, the monitoring team would start an investigation. We will use a significance level of 5%. The monitoring team and investigators would decide to discontinue or modify interventions should the investigation reveal that the increase in mortality is likely due to this study.

#### Follow-up of adverse events

Severe maternal and neonatal outcomes due to delayed and/or inappropriate care could occur as a result of this study. Neonatal deaths in the target areas are routinely reported through the health information system. Those data are available online.

#### Protocol amendments

If protocol modifications were needed, investigators would immediately report those to the ethics committee/institutional review board and explain the modifications to the trial participants and collaborating partners (Lao MoH, WHO). Amendments to the protocol will be updated in the trial registry as well.

#### Access to data

Investigators will have access to the final trial dataset. The monitoring team and REC/IRB will have access to data if ethical considerations arise. Investigators will share the anonymised data with collaborating organizations, the Lao MoH and the WHO when requested.

#### Ancillary and post-trial care

This study may impose time and mental burdens on participants, especially those in the intervention group because district hospitals in the intervention group must conduct self-managed continuous monitoring activities quarterly. However, EENC is expected to be routinely provided at every birth in Lao PDR. Therefore, introduction of EENC itself will not require participants to do additional work. We anticipate that the burden of additional questionnaires and exams on participants will be trivial. We will consider implementing refresher EENC coaching if there is an increase in neonatal deaths in district hospitals and if health workers have a lower level of knowledge and skills for EENC at the end of this study compared to the baseline.

#### Dissemination policy

Study results will be promptly shared with trial participants, provincial and district offices/hospitals, the Lao MoH and the WHO. Results will be reported at academic conferences nationally and internationally. The results will also be disseminated in academic publications. Authorship eligibility principally follows recommendations of the International Committee of Medical Journal Editors (ICMJE) [31].

We will provide the Lao MoH with feedback on the most effective method for maintaining quality of EENC. We will work with the WHO to extract lessons learnt for improved EENC guidelines in the Western Pacific Region.

## Discussion

Maintaining quality of newborn care is essential to prevent neonatal deaths; however, supervision of medical professionals imposes a heavy burden on the health system in resource-limited countries. Self-managed continuous monitoring of newborn care could be an alternative, sustainable and cost-effective method to address this issue. This trial is remarkable in terms of its novelty, timing and impact on the country’s policy. As far as we know, this is the first trial to test the effectiveness of self-managed continuous monitoring to maintain quality of EENC at the district level in a resource-limited country. This trial is very timely because the Lao MoH have decided to scale up EENC to the district level in 2017 and beyond. The EENC will be initially implemented in the targeted areas of this trial. Therefore, trial findings could change the strategic direction of EENC quality maintenance in other provinces in the country. Ensuring EENC of high quality on a large scale will reduce preventable neonatal deaths and minimise health disparities. As this trial is conducted in a resource-limited country, the results could influence activities in other countries. In conclusion, this trial will provide timely and essential information on an effective, practical and sustainable intervention aimed at maintaining quality of EENC in the wider area of the country and region. A limitation of this study is that given that the intervention consists of several elements, unravelling the effect of each element and its mechanism in improving quality of care could be difficult. Another limitation is that this study does not focus on respectful attitudes towards service takers. As respectful care is an important element of quality, further investigation on improvement of health workers’ attitudes would be necessary.

## Additional files

Additional file 1: Guideline on self-managed continuous monitoring, Ministry of Health, Lao PDR. (PDF 684 kb)

Additional file 2: Questionnaire on determinants to perform Early Essential Newborn Care. (DOCX 22 kb)

Additional file 3: Written test on Early Essential Newborn Care. (PDF 1653 kb)

Additional file 4: Simulation test on Early Essential Newborn Care. (DOCX 24 kb)

Additional file 5: Checklist of hospital environment facilitating implementation of Early Essential Newborn Care. (DOCX 55 kb)

Additional file 6: General Information Questionnaire. (DOCX 20 kb)

Additional file 7: Informed consent form. (DOCX 23 kb)

## Abbreviations

EENC: Early Essential Newborn Care
ICC: Intra-cluster Correlation Coefficient
ICMJE: International Committee of Medical Journal Editors
ITT: Intention to treat
Lao PDR: Lao People’s Democratic Republic
LOCF: Last observation carried forward method
MI: Multiple imputation method
MoH: Ministry of Health
RCT: Randomized controlled trial
UNICEF: United Nations Children’s Fund
WHO: World Health Organization
WPRO: Western Pacific Regional Office of the World Health Organization

## References

[1] UNICEF, WHO, World bank, et al*.* Levels & Trends in Child Mortality. New York: 2015. doi:10.1371/journal.pone.0144443.

[2] World Health Organization. Child Mortality, World Health Statistics 2016. Geneva: WHO: 2016.

[3] World Health Organization Regional Office for the Western Pacific (2016). First biennial progress report.

[4] World Health Organization Regional Office for the Western Pacific. WPRO | Fact sheet on child health. 2015.http://www.wpro.who.int/mediacentre/factsheets/docs/fs_201202_child_health/en/. Accessed 11 Nov 2017.

[5] Sudfeld CR, Fawzi WW, Collaboration TGB of DC and AH (2017). Importance of innovations in neonatal and adolescent health in reaching the sustainable development goals by 2030. JAMA Pediatr.

[6] E, Lawn J, Cousens S, Zupan J. 4 million neonatal deaths: when? Where? Why? Lancet. 2005;365:891–900. 10.1016/S0140-6736(05)71048-5 15752534

[7] Sankar MJ, Natarajan CK, Das RR (2016). When do newborns die? A systematic review of timing of overall and cause-specific neonatal deaths in developing countries. J Perinatol.

[8] Helping Babies Breathe Lessons learned guiding the way forward A 5-year repor t from the HBB Global Development Alliance.

[9] World Health Organization Regional Office for Western Pacific (2014). UNICEF. Action plan for healthy newborn infants in the western Pacific region (2014–2020).

[10] World Health Organization (WHO) (2014). Early essential newborn care clinical practice pocket guide early essential newborn care clinical practice pocket guide.

[11] Pammi M, Dempsey EM, Ryan CA (2016). Newborn resuscitation training Programmes reduce early neonatal mortality. Neonatology.

[12] Musafili A, Essén B, Baribwira C (2013). Evaluating helping babies breathe: training for healthcare workers at hospitals in Rwanda. Acta Paediatr Int J Paediatr.

[13] Dempsey E, Pammi M, Ryan AC, et al. Standardised formal resuscitation training programmes for reducing mortality and morbidity in newborn infants. Cochrane Database Syst Rev Published Online First: 4 September 2015. 10.1002/14651858.CD009106.pub2. 10.1002/14651858.CD009106.pub2 PMC9219024 26337958

[14] Ersdal HL, Vossius C, Bayo E (2013). A one-day ‘helping babies breathe’ course improves simulated performance but not clinical management of neonates. Resuscitation.

[15] World Health Organization (2016). Global strategy on human resources for health: workforce 2030.

[16] International Confederation of Midwives. Essential competencies for basic midwifery practice2010 Revised 2013. 2016.

[17] Bosch-Capblanch X, Liaqat S, Garner P. Managerial supervision to improve primary health care in low- and middle-income countries (Review). Cochrane Libr Published Online First: 2011. 10.1002/14651858.CD006413.pub2. 10.1002/14651858.CD006413.pub2 PMC6703669 21901704

[18] Hill Z, Dumbaugh M, Benton L (2014). Supervising community health workers in low-income countries--a review of impact and implementation issues. Glob Health Action.

[19] Bailey C, Blake C, Schriver M (2016). A systematic review of supportive supervision as a strategy to improve primary healthcare services in sub-Saharan Africa. Int J Gynecol Obstet.

[20] Ministry of Health Lao PDR, World Health Organization (2015). Success factors for Women’s and Children’s health Lao PDR.

[21] World Health Organization (2011). Sexual and reproductive health Core competencies in primary care.

[22] Department of Planning and International Cooperation. Ministry of Health Lao PDR. National Health Statistic Report 2016. Vientiane, Lao PDR: 2016.

[23] Lao Statistics Bureau. Results of population and housing census 2015. Vientiane: Ministry of Planning andInvestment of Lao PDR; 2015.

[24] Indochina Research Limited. Facility Assessment for Reproductive Health Commodities and Services in Lao PDR: 2014 Survey Report Vientiane: 2014.

[25] Ministry of Health, World Health Organization Country Office Lao PDR. Service Availability and Readiness Assessment Survey Report 2014 Lao PDR Vientiane: 2014.

[26] Pagel C, Prost A, Lewycka S (2011). Intracluster correlation coefficients and coefficients of variation for perinatal outcomes from five cluster-randomized controlled trials in low and middle-income countries: results and methodological implications. Trials.

[27] Ajzen I (1991). The theory of planned behavior. Orgnizational Behav Hum Decis Process.

[28] Ajzen I. Constructing a theory of planned behavior quesionnaire. 2006:1-12.

[29] Measurement Instrument Database for the Social Sciences. Theory of Planned Behaviour Questionnaire. http://www.midss.org/content/theory-planned-behaviour-questionnaire. Accessed 15 May 2017.

[30] Francis JJ, Eccles MP, Johnston M (2004). Constructing questionnaires based on the theory of Plannd behaviour-a manual for health services researchers. Newcastle upon Tyne.

[31] International Committee of Medical Journal Editors. Recommendations for the Conduct, Reporting, Editing, and Publication of Scholarly Work in Medical Journals. 2016. 25558501

