# Supplementary material for: Study protocol of a cluster randomized controlled trial to evaluate effectiveness of a system for maintaining high-quality early essential newborn care in Lao PDR
Source: BMC Health Serv Res. 2018 Jun 25;18:489. doi: 10.1186/s12913-018-3311-7 (PMC6019299; doi:10.1186/s12913-018-3311-7)
Supplement: Supplementary file 1 — Guideline on self-managed continuous monitoring, Ministry of Health, Lao PDR. (PDF 684 kb) [file 12913_2018_3311_MOESM1_ESM.pdf]

Establishing Quality Improvement for Early Essential Newborn Care in Hospitals

Health Facility Strengthening Guide Adapted for Lao PDR

WORLD HEALTH ORGANIZATION

REGIONAL OFFICE FOR THE WESTERN PACIFIC

World Health Organization
Western Pacific Region

Introduction

Early essential newborn care (EENC) is a package of interventions provided to the mother and newborn during childbirth until 3 days later. EENC interventions are simple, low cost and have been demonstrated to be effective in preventing newborn deaths from the most common causes. EENC also aims to eliminate outdated, harmful or ineffective practices that remain widespread.

EENC implementation begins with coaching staff who provide services to mothers and babies. Within hospitals, selected coached staff then form an 'EENC Team', which is responsible for quality improvement. This guide describes what is needed to establish EENC quality improvement in hospitals.

This guide is also used by external monitoring teams to provide data on national progress to inform annual planning. Evaluators, using the same methods as for self-monitoring, with some additional tools, determine the status of EENC implementation at health facilities.

Purpose: To provide the EENC Team with a practical approach to assess and improve EENC quality in their hospital (self-monitoring) and in other hospitals in the country (external monitoring).

Expected Outcome: After an initial joint monitoring visit, a team WITHIN each hospital will be able to conduct self-monitoring using the tools without external support. (see timeline below)

Participants:

- Hospital Director
- head of OBGY ward
- Head of Paediatric Ward
- Delivery ward staff (doctor, midwife, nurse)
- NICU staff (doctor, nurse)_
- Hospital Administration
- Infection control

Hospital director, head of Pediatric ward and all staff in the delivery ward and NICU should participate in the committee meeting on the second day.

Timing:

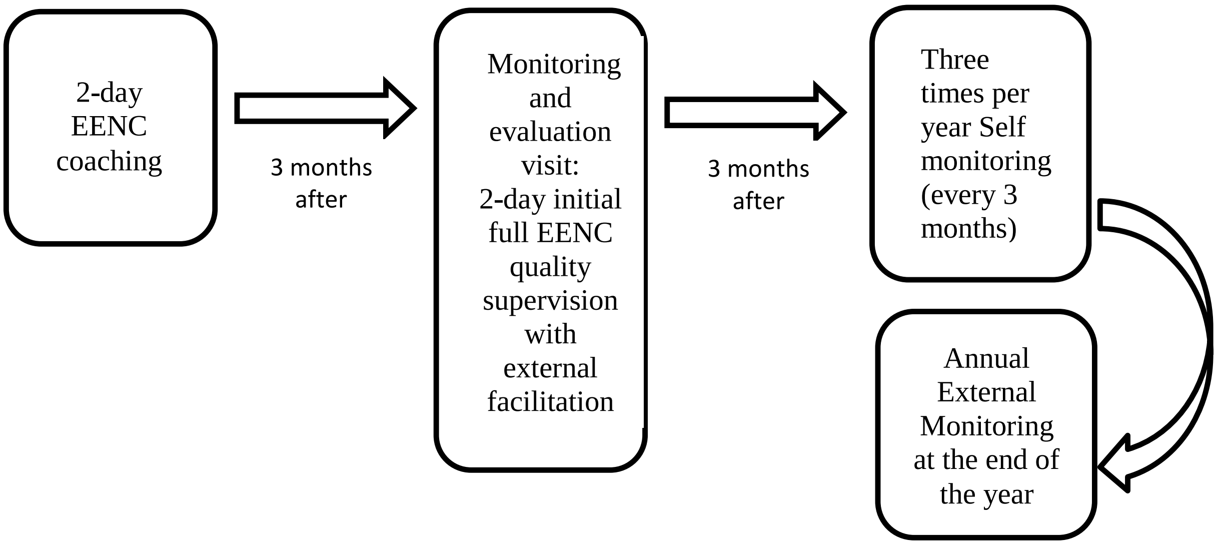

* the EENC Team may decide to conduct assessments more frequently depending on the status and quality of EENC practice.

Method: See Table 1. Contents of the Full EENC Quality Assessment.

Materials needed for initial full EENC quality assessment[1]:

1. This guide: Establishing Quality Improvement for Early Essential Newborn Care in Hospitals (1 per participant).
   Please note: Chapter I should be used for self-monitoring and external monitoring
   Chapter II should be used only for external monitoring.
2. Flipcharts (10)
3. Markers (10)

Model agenda for EENC 2 days Quality Assessment for self- monitoring

- The table below is a list of the tools available to do the Quality Assessment
  - Please try to use all checklists to provide feedback within your EENC team
- A report of your EENC Quality assessment needs to be submitted to the Ministry of Health, Department of Healthcare, three times per year. The template is in Annex 1. The results of the checklists do not need to be submitted, they are meant to be utilized as a feedback tool only.

**Time**	**Purpose**	**Tasks under the action**	**Tools used (Outcomes)**	**People involved**
**Day 1**		**Introduction to the tools**	**Booklet**	
Morning and afternoon	Review EENC clinical practice	Introduction		Staff from the delivery room and NICU
		Exit interviews with postpartum mothers	Chapter I: Checklist 1	
		Chart review of mother who received exit interview	Chapter I: Checklist 2	
		Observation of delivery practice and environmental hygiene	Chapter I: Checklist 3a, 3b,	
		Observation of environmental hygiene	Chapter II: Checklist 4	
		Medications	Chapter II: Checklist 5	
		Hospital policy	Chapter II: Checklist 6	
**Day 2**			**Booklet**	**All**
Day 2<br>8:00-10:00	Summarize data.	• Complete flipcharts	Flipcharts from each checklist	
10:00-12:00	Identify areas for improvement. Actions to Solve the Areas for Improvement	• Identify EENC strengths<br>• Identify and prioritize EENC areas for improvement<br>• Develop action steps<br>• Assign responsibility and timeline	Table 1a<br><br>Table 2a	
13:30-16:00	EENC committee meeting	• Each staff present their responsible area<br>• Comments/Feedback		

1. CHAPTER I: Exit interviews, Chart reviews, Observation of delivery

INTRODUCTION TO TRAINING FOR SELF MONITORING (MODULE 3)

- Handout the booklet to every participant. PLEASE USE ONLY CHAPTER I OF THIS HANDBOOK
- Explain purpose and rationale of the visit:
  - Monitor hospital's progress in EENC externally for the first visit
  - An opportunity to give feedback about how your hospital is performing in EENC clinical practice
  - Teach your hospital how to conduct EENC **self-monitoring** in the future
  - Make clear expectations for EENC self-monitoring in your hospital
- Read through every checklist and clarify misunderstandings
- Allocate tasks to every participant:
  - Postpartum interviews
  - Chart Reviews
  - Observation of Delivery
  - Attendance at committee meeting
- Arrange when to meet in the large group
- Allocate flipcharts/other for recording the results of the checklists for discussion in the EENC committee meeting

1.1 Exit Interviews with Postpartum Mothers: Term Babies and Preterm or Low Birth Weight Babies

Instructions

On the job training: Supervising facilitator to do the first interview as an example. After this the local hospital member does the next interview under supervision from the supervising facilitator.

1. Ten interviews are conducted with postpartum women of term or preterm (<37 weeks) or low birth weight (LBW) (<2000g) babies before discharge.
2. Divide the group into pairs, with each pair conducting at least two exit interviews - one person interviewing and the other recording. Interviews should be completed before women are discharged.
3. Identify a room away from patients and staff for conducting interviews. If this is not possible, locate a quiet corridor or public area where the conversation can be more private.
4. Select mothers using these criteria:
   - Delivered at least 3 hours prior to the interview.
   - A mix of women with normal vaginal deliveries, assisted deliveries, caesarean sections (when present) and babies in the NICU.
   - Have not been admitted for abortion, or had a stillbirth or newborn death.
   - If 10 or fewer postpartum mothers meeting the selection criteria are available, select all mothers. If more than 10 postpartum mothers meeting the selection criteria are available, please attempt to interview women to represent each of the following: term, pre-term birth, LBW baby.
5. Obtain informed verbal consent. State, "We are trying to understand your delivery experience so that we can help improve care for women. Everything you say here will be kept confidential, meaning no one will know you said it. Anytime you want to stop, you may. Your care will remain the same. Do you agree to do this interview?" Record informed consent given.
6. Conduct exit interview and record in a notebook.
   - State: "We would like to start by asking you to describe what happened to you from the moment you went in labor until now."
   - Probe: the silent probe (i.e., maintaining silence even after you feel uncomfortable) with head nods is very effective in allowing women to tell their story. This can be followed by: "so the first thing that happened was… [repeat what was said], what happened next." Keep probing to fill in the details.
   - Write down the "story" of her labor and delivery. Ask her to tell it in her own words. Record the story in a notebook, making special note of responses to questions in checklist 1a. If by the end of her telling her story, the mother does not tell you specific details spontaneously, then use the questions in the Checklist 1a.
   - The questions in the Checklist 1a should not be used to conduct the interview until the mother has finished telling the story.
7. Record information in **Checklist 1a** indicating Y (yes), N (no) or as otherwise instructed in the questions**. Summarise in checklist 1b.** If question is not applicable (example: pre-term baby questions for term babies), please indicate N/A

Checklist 1. Exit interviews with Postpartum Mothers

**Question**<br>Answer the questions with Y (= yes) or N (= no)	**Mother number**										**n*/N ****	**(%)**
	**1**	**2**	**3**	**4**	**5**	**6**	**7**	**8**	**9**	**10**		
1. Verbal informed consent obtained												
2. Mode of delivery (V = vaginal, CS = Caesarean section)											**n of V:**<br>**n of CS:**	**% of V:**<br>**% of CS:**
3. Age of the baby (Hours)												
4. During childbirth,<br>a. Was the mother allowed to sit, stand or lay in the position she wanted?												
b. Did the mother have a companion of her choice?												
c. Was the mother encouraged to eat and drink?												
d. Did anyone push down on the mother’s belly? (Fundal pressure)												
e. Was the mother encouraged to walk around?<br>f. Was the mother encouraged to urinate?												
5. Was the baby bathed?												
a. How long after birth (<24h, >24h)											n ≥24 h	% >24h
6. Was the baby placed in skin-to-skin contact with the mother? If yes,												
a. How long after birth (<1, 1-10, 11-59, ≥60 min)?											n <1 min	% <1min
b. How long did the baby remain in <u>uninterrupted</u> skin-to-skin contact before being separated from the mother for any reason? (<10, 10-29, 30-59, 60-89, ≥90 min)											n ≥ 90 min	% > 90 min
c. Had the baby completed the first breastfeed (attached, deep sucking) before being separated from the mother?												
d. Why was the baby separated from the mother? (needed resuscitation/ NICU admission, for routine care)											Reasons	
e. Did the baby receive a) immediate skin-to-skin contact, b) no separation for at least 90 minutes and c) until the first breastfeed was completed? (answer Y only if a)<1 min, b) ≥ 90 min and c) = Y)												
7. Did the baby stay with the mother the entire hospital stay (rooming in)?												
8. Is the mother breastfeeding? If yes,												
a. How long after birth did the baby first breastfeed? (<15, 15-90, >90 min) (attached, deep sucking)											n (15-90 min) =	%
b. How long did the baby breastfeed the first time?											n > 15 min =	%
c. Since delivery was her baby fed anything other than breast-milk?											n (# No) =	
d. Did the baby receive a) early (within 15 – 90 min) and b) exclusive breastfeeding (Answer Y only if both a) = 15 - 90 min and b) = Yes)												
9. If the baby has been fed anything other than breastmilk, what is being given? (water, formula, other)											Fluids given	

**Question**<br>Answer the questions with Y (= yes) or N (= no)	**Mother number**										**n*/N**<br>****	**(%)**
	**1**	**2**	**3**	**4**	**5**	**6**	**7**	**8**	**9**	**10**		
10. Has the baby been fed anything from a bottle?												
11. Was anything applied to the cord stump?												
a. If yes, what was applied? (alcohol, triple dye, other)											Substances:	
12. Does the mother know at least two benefits of breastfeeding? (Ask the mother directly)												
13. Has the mother received counselling on breastfeeding since birth? (for example: feeding cues, positioning of the baby, signs of attachment, how often to feed and how long to feed, importance of only breast milk and no other foods or fluids)												
14. Has the mother been given any promotional products sponsored by a baby food company (bottles, dummies, clothing, infant formula or any other branded material)?												
13b. If yes, who was she given it by?												
13. Does the mother have any infant formula with her?												
**If mother of preterm or low-birthweight baby:**												
14. Is the mother giving breastmilk not by breast? If Yes,												
a. How is the milk being given? (cup, spoon or stomach tube)												
b. How often is she expressing breastmilk? (frequency in hours)											n > 8 times per 24 h =	%
15. For preterm babies, how many times was breast milk given in the last 24 hours?												
16. If something else than breast milk was given,												
a. If yes, What was given?												
b. Was it given before the first breastfeed?												
17. Is the baby in STS contact in KMC position? [a.]												
18. Was the baby in KMC?<br>If Yes, in the past 24 hours,												
a. Was KMC applied ≥ 18 hours												
b. Were there any separations for more than 30 minutes?												

n = total number of "Y" (Yes) responses unless otherwise specified
**N = total number of mothers interviewed
[a.] Stable babies <37 weeks and/or < 2.5 kg, in skin-to-skin contact with mother at the time of review

1.2. Chart Review of Postpartum Mothers who Received an Exit Interview: ***Term Babies and Preterm or Low Birth Weight Babies***

Instructions

On the job training: Supervising facilitator to do the first chart review as an example, After this, the local hospital member does the next chart review under supervision from the supervising facilitator. (The local hospital staff member should write down the results)

1. In pairs, use the identification numbers of the mother and baby to identify the chart of women who already received an exit interview. If mothers' charts are separate from that of the baby, it may be necessary to review both to complete the chart review.

2. In pairs, complete **Checklist 2a. Chart Review of Postpartum Mothers who Received an Exit Interview.** If data are not recorded in the chart the response is 'N'.

3. If question is not applicable (example: pre-term baby questions for term babies), please indicate N/A

4. In plenary, tally the results in **Checklist 2b. Summary Table: Chart Review of Postpartum Mothers who Received an Exit Interview.**

Checklist 2. Chart Review of Postpartum Mothers who received an Exit Interview

**Question**<br>Answer questions with Y (= yes), N (= no) or Not recorded (=NR)	**Mother number**										**n*/N ****	**%**
	**1**	**2**	**3**	**4**	**5**	**6**	**7**	**8**	**9**	**10**		
1. Identifying information of mother and baby												
2. Birthweight (in grams)												
3. Gestational age (in weeks)												
4. Were syphilis test results from ANC written in the record? If Yes,												
a. Was the test positive?												
b. Were actions taken in the antenatal period to address the positive syphilis test?												
5. Were HIV test results from ANC or point of care rapid test written in the record?												
6. Was a partograph completed correctly? [a]												
a. If partial or no, specify reason.											Main reason	
7. Was artificial rupture of membranes (amniotomy) done?												
8. Was the mother's labor augmented with oxytocin?												
a. If Yes, were appropriate criteria used? [b]												
9. Was the baby delivered by caesarean section?												
a. If Yes, were appropriate criteria used? [c]												
10. Was an episiotomy done?												
a. If yes, were restricted criteria for episiotomy used? [d]												
11. Were uterine tone, bleeding volume, fundal height, temp, pulse and BP of the mother all recorded in chart within 1 h of delivery?												
12. Was the mother's urine void recorded within 6h after delivery?												
13. Was IM oxytocin given after delivery?												
14. Was routine eye care given within 90 minutes of birth? [e]												
15. Was vitamin K given between 90 min and 6 h of birth? [e]												
16. Was hepatitis B vaccine given within 24 h of birth? [e]												
17. Was BCG vaccine given within 24 h of birth? [e]												
For pre-term and low birth weight babies only:												
18. Mother 24 - 34 weeks of gestation meeting criteria received antenatal corticosteroids within 1 h of arrival? [f]												
19. Mother < 32 weeks of gestation received magnesium sulfate within 1 h of arrival?												
20. For preterm and LBW babies: Were baby vital signs measured at least 4 times in the last 24 hours?												

n = total number of "Y" (Yes) responses unless otherwise specified
**N = total number of mothers interviewed

[a] P=partial may be applicable. If the assessor is unable to determine whether the partograph has been filled correctly, the answer should be validated with the overseeing staff member/attending physician. Please refer to Emoc guidelines for correct completion of partograph.
[b] The following are documented in the partograph or chart before starting augmentation: normal fetal heart rate AND cervical dilatation crossing the alert line AND inadequate uterine contraction.
[c] At least 1 of the following indications for CS documented: ante-partum haemorrhage due to major placenta previa or placental abruption with fetal distress; presence of severe preeclampsia (within 24 hours)or eclampsia (within 12 hours) AND an unfavorable cervix; failed induction; failure to progress in labour based on partograph (tried to induce or augment labour with oxytocin and ARM (artificial rupture of membranes) but with no labour, or obstructed labour); fetal distress where vaginal delivery is remote; transverse lie in labour; cord prolapse with live fetus and delivery remote;

chorioamnionitis - associated with poor progress in labour; previous caesarean delivery and indications that vaginal birth after caesarean was not feasible.

[d] One or more of the following documented: abnormal progression of labour; non-reassuring fetal heart rate pattern; vacuum or forceps delivery; shoulder dystocia; poorly healed tears.

[e] If timing of administration is not specified, indicate 'Y' and 'TNS' (time not specified).

[f] Gestational age can be accurately assessed; preterm birth is imminent; no clinical evidence of maternal infection.

1.3 Observations of Delivery Practices

Instructions

Observations of delivery practices

Supervising facilitator to supervise local hospital staff member to observe the delivery and complete the checklist.

1. Ask delivery and operation room staff to notify the group of pending deliveries and caesarean sections.

2. In pairs, move about to get a clear view without obstructing the birth attendant(s), speaking or intervening.

3. Observe the same delivery, record findings individually on **Checklist 3a** or **3b** as correctly done (y = yes), done but incompletely (p = partial) or not done or done incorrectly (n = no). If a practice is not applicable or not assessed, indicate N/A, and provide details in the 'Comments' column.

4. After each observation, score the checklist: 2 points for Yes, 1 point for Partial and 0 points for No. The maximum possible score for delivery of a breathing baby (Checklist 3a) is 42 and non-breathing baby (Checklist 3b) is 60. Upon completion, compare findings in the pair and reconcile differences.

5. Give feedback to staff at the end of the delivery away from the mother. Provide positive feedback first and then describe areas for improvement.

Checklist 3a. Delivery practice for the Breathing Baby (please write on a flipchart)

Date: _______________ Location: _______________

Observation conducted by: _______________________________

**Activity**<br>(Answer Y = Yes, N = No, P = Partial, N/A = not applicable or not assessed)	**Observation #**					**Summary**		
	**1**	**2**	**3**	**4**	**5**	**Yes (n)**	**No (n)**	**P (n)**
Mode of delivery (V = vaginal, C = caesarean section)								
**Pre-Birth Preparation:**								
1. Checked room temperature; turned off fans and/or air conditioning								
2. Washed hands before touching any delivery area surfaces and handling equipment								
3. Placed dry cloth on abdomen								
4. Prepared the newborn resuscitation area								
5. Checked if newborn ambu bag and mask are functional								
6. Prepared and checked suction bulb								
7. Washed hands before gloving for delivery								
8. Wore two pairs of sterile gloves (if same attendant handles the cord)[1]								
9. Arranged forceps, cord clamp/ties in easy to use order								
**Immediate Postpartum/Newborn Activities:**								
10. Called out time of birth (hours, minutes, seconds)								
11. Drying started within 5 sec after birth?<br>*Answer <5 sec (2 points), 5-10 sec (1 point), >10 sec (0 points)								
12. Dried the baby thoroughly (wiped the eyes, face, head, front, back, arms and legs)								
13. Removed the wet cloth								
14. Baby was in direct skin-to-skin contact								
15. Covered baby's body with cloth and head with a hat								
16. Checked for a second baby								
17. Oxytocin IM given to mother within 1 minute								
18. 1st pair of gloves removed[1]								
19. Cord pulsations checked before clamping, clamped after cord pulsations stopped (usually 1 – 3 minutes)								
20. Clamp/tie placed at 2 cm, forceps at 5 cm from umbilical base								
21. Delivered placenta								
22. Counseled mother on feeding cues (drooling, mouth opening, tonguing/licking, rooting, biting hand, crawling, etc)<br>*Answer 1-2 mentioned (1 point), >2 mentioned (2 points)								
**Total Score: (# Yes x 2 + # Partial x 1)**<br>(maximum score possible = 42)								

[1] If the mode of delivery is caesarean section or a separate birth attendant is available to handle the cord, indicate N/A here but enter or score the practice as a 'Yes' with two points even if only a single set of sterile

gloves is worn by the health worker that delivers the baby. However, subtract 2 points if the gloves handling the cord were in any way not sterile.

Checklist 3b. Delivery practice for the Non-Breathing Baby (please write on a flipchart)

Date : _______________ Location: _______________

Observation conducted by: _______________________________

**Activity** (Answer Y/N/P, Y = Yes, N = No, P = Partial, N/A = not applicable or not assessed)	**Observation #**					**Summary**		
	**1**	**2**	**3**	**4**	**5**	**Yes (n)**	**No (n)**	**P (n)**
Mode of delivery (V = vaginal, C = caesarean section)								
**Pre-Birth Preparation:**								
1. Checked room temperature; turned off fans								
2. Washed hands before touching any delivery area surfaces and handling equipment								
3. Placed dry cloth placed on abdomen								
4. Prepared the newborn resuscitation area								
5. Checked if newborn ambu bag and mask are functional								
6. Prepared and checked the suction bulb								
7. Washed hands before gloving for delivery								
8. Wore two pairs of sterile gloves (if same attendant handles the cord)[1]								
9. Arranged forceps, cord clamp/ties in easy to use order								
**Immediate Postpartum/Newborn Activities:**								
10. Called out time of birth (hours__, minutes__, seconds__)								
11. Drying started within 5 sec after birth? *Answer <5 sec (2 points), 5-10 sec (1 point), >10 sec								
12. Dried the baby thoroughly (wiped the eyes, mouth/nose, face, head, front, back, arms and legs)								
13. Removed the wet cloth								
14. Put baby in direct skin-to-skin contact								
15. Covered baby's body with cloth and head with a hat								
16. Determined the baby was gasping or not breathing								
17. Called for help and informed the mother								
18. Removed first pair of gloves[1]								
19. Quickly clamped and cut cord								
20. Moved baby to resuscitation area								
21. Covered baby quickly during after transfer								
22. Positioned head correctly to open airways								
23. Applied face mask firmly over chin, mouth & nose								
24. Gained chest rise within 1 minute of birth[2]: Minutes___ : Seconds ___								
25. Squeezed bag to give 30-50 breaths per min								
26. Maintained good chest rise throughout or took steps to improve ventilation								
27. After baby breathing well, stopped ventilation								
28. Returned to skin-to-skin contact, covered baby								
29. Checked for another baby								
30. Gave oxytocin IM to the mother								
31. Delivered placenta								
32. Counseled mother that baby is ok and on feeding cues								
**Total Score: (# Yes x 2 + # Partial x 1) [**maximum = 60][3]								

[1] If the mode of delivery is caesarean section or a separate birth attendant is available to handle the cord, indicate N/A here but enter or score the practice as a 'Yes' with two points even if only a single set of sterile

gloves is worn by the health worker that delivers the baby. However, subtract 2 points if the gloves handling the cord were in any way not sterile.
[2] Only scored as 'Yes' or 'No', no partial.
[3] Deduct 5 points if resuscitation is performed when it is not required: if the baby is not breathing but has muscle tone and a grimace, and then is not dried appropriately (either not immediately, not thoroughly or not at al

1.4 Identify and Prioritize EENC Strengths and Areas for Improvement for EENC

Instructions

- Call all the participants to a meeting including:
    - Hospital Director
    - head of OBGY ward
    - Head of Paediatric Ward
    - Delivery ward staff (doctor, midwife, nurse)
    - NICU staff (doctor, nurse)_
    - Hospital Administration
    - Infection control

Process of data analysis and planning actions

A. Review action plan from the last monitoring (Use Table 2 from the last monitoring)
   1. Ask provincial / district staff to report progress (Record in the Table 2 "Status / Date")
   2. Review repeated problems and reasons
   3. Ask the provincial / district team to discuss and agree on the actions to be taken with timeframe and responsibilities (record in Table 2)
      * Repeated problems have reasons of not implementing, so they cannot copy the same actions. They need to agree on realistic actions that they can act upon.

B. Review exit interview of mothers
C. Review chart reviews
D. Review direct observation
E. Review environmental hygiene (for external monitoring only)
F. Review medicines and supplies (for external monitoring only)

[Repeat the 1-5 following process for II to VI]

1. Ask provincial / district staff to report the result
2. Ask the provincial / district team to identify strengths (record in Table 1)
3. Ask the provincial / district team to discuss and agree on which areas for improvement to prioritize (record in Table 1)
4. Ask the provincial / district to discuss and agree on bottlenecks
5. Ask the provincial / district team to discuss and agree on actions to be taken with timeframe and responsibilities (record in Table 2)* Only if there are issues that cannot be solved within the facility, record in the column "Request for support"

Tips for the external facilitators:

1. Regulate the step-by-step process (Eg. If the provincial team jumped the discussion from selecting priority problems to solution, you need to bring them back)
2. Facilitate the discussion to be specific based on the data, and redirect it when they state bottleneck or solution that are general (We often observe general reasons and solutions that could come out without having specific data.)
3. Not tell them what are the problems, bottlenecks, solutions are: they should come from

the team themselves

Table 1a. Identifying and Prioritizing Strengths and Areas for Improvement for EENC

	Strengths	Areas for Improvement
Interviews		
Chart reviews		
Observation of delivery		

Table 2a: Priority Actions for Improving EENC

Priority Actions	Person-responsible	Time	Status/ Date
Pending issues:<br>•<br>•			
Interviews:<br>•<br>•<br>•			
Chart reviews:<br>•<br>•<br>•			
Observation of delivery:<br>•<br>•<br>•			
**Request to Province/Central**<br>*** Most of the actions need to be taken by your own facility. This request is for an exceptional case only if you have crucial issues that cannot be solved in your facility.**			

2. Chapter II: Review of environmental hygiene, availability of Key Medicines and Supplies and Hospital policies

Please note, these checklists should only be completed during external monitoring.

2.1 Observation of environmental hygiene

Checklist 4. Environmental Hygiene: Delivery Room, Recovery Room, Neonatal Care Unit and Postnatal Care Room
REMOVE ALL DIFFERENT ROOMS

**Question**	**Delivery Room (s)**	**Recovery room (s)**	**Neonatal Care Unit**	**PNC Room (s)**	**Comments**
**Hand washing facilities and toilets for patients[1]:**					
1. What is the total number of rooms? (N)					
2. Rooms have at least one sink for washing hands available for use in each room?[2] (n/N)					
3. What is the total number of sinks? (N)					
4. How many sinks:					
b. are clean? (n/N)					
c. have continuous clean, running water[3]? (n/N)					
d. have soap[4] available? (n/N)					
e. have at least one method to dry hands: single-use towel, hand drier, reusable sterile towels					
5. Rooms have adequate sink hand washing facilities available (n/N) (n= Y if each room has at least one sink and 4. a.-c. = 100% for all sinks in the room).					
6. Rooms have at least one bottle of alcohol gel/hand rub available for use in the room? (n/N)					
7. Rooms have adequate sink hand washing facilities AND alcohol hand gel available for use in the room (n/N). (n= Y if 5. = Y and 6. = Y).					
8. How many toilets exist for patients?					
8a. Are clean?					
8b. Are functioning?<br>8c. Are not further than 30m from all users?<br>8d. Are not further than 5m from hand washing facility?					
**Newborn Resuscitation area:**					
9. How many delivery beds ?<br>10. How many delivery beds have a resuscitation area available within 2 m?					
11. How many resuscitation areas are available?					
12. How many resuscitation areas:					
a. are clean and dry?					
b. have newborn ambu bag and mask available?					
**Promotion of baby food company products**					
13. Are baby food company materials visible (posters, brochures, stickers, painted walls, etc)					
14. Are there points of sale inside the grounds of the hospital? (minimart or direct staff selling)					
15. Is there infant formula visible in the post-partum wards?					

16. Have health workers been given any gifts from baby food companies (pens, notebooks, cups, pencil holders, meals etc)					

[1] To undertake a complete hand hygiene assessment, see 'Hand Hygiene Self-Assessment Framework' (WHO, 2010)
[2] If more than one room is available in a category, report availability in each room separately. Note if alcohol gel/hand rub is available for staff use but not for use by patients and families.
[3] A water supply that is either piped or from onsite storage, with appropriate disinfection, meeting appropriate safety standards for microbial and chemical contamination.
[4] Soap: detergent-based products that contain no added antimicrobial agents or may contain these solely as preservatives. It may be in various forms including bar soap, tissue, leaf and liquid preparations.

2.2 Review of availability of key medicines and supplies for EENC

Instructions

- Review the list of medicines and supplies by direct observation – staff who work in ANC, delivery, postnatal care and neonatal care areas are often familiar with the availability of essential medicines, equipment and supplies and can help identify where medicines and supplies are stored and answer key questions.

- If EENC team members are unsure of the status of some medicines or supplies, determine who should be consulted to determine the status. This may include staff from the relevant section or the hospital pharmacy.

- Complete Checklist 4 (next page).
    - The WHO definition of "Normal storage conditions" is: "Storage in dry, well-ventilated premises at temperatures of 15-25 °C or, depending on climatic conditions, up to 30 °C"
    - Note items not available on the day of the review and those that have had stock-outs in the previous 12 months. Note problems with storage or functionality of equipment.
    - Note whether stock records are available for all items.

Checklist 5. Review of availability of key medicines and supplies for EENC

	Available on the day of the review? Y or N	Stock condition? No expired drugs? Equipment functional?	Stock records? (Y or N)	# Stock-outs in last 12 months
1. Magnesium sulfate for severe pre-eclampsia and eclampsia, and fetal neuroprotection if gestational age <32 weeks		☐ Normal storage [a]<br>☐ No expired drugs		
2. Oxytocin for IM and parenteral use – immediately postpartum and for control of hemorrhage		☐ 2°C -8°C<br>☐ Protected from light [b]<br>☐ No expired drugs		
3. Corticosteroids for women of 24 – 34 weeks of gestation at risk of preterm delivery [c]		☐ Normal storage [a]<br>☐ Protected from light [b]<br>☐ No expired drugs		
4. Injectable antibiotics for management of newborn sepsis		☐ Normal storage [a]<br>☐ Protected from light [b]<br>☐ No expired drugs		
5. Antibiotics for preterm pre-labour rupture of membranes[d]		☐ Normal storage [a]<br>☐ Protected from light [b]<br>☐ No expired drugs		
6. Syphilis test kits				
7. HIV test kits				
8. First line ART regimen: tenofovir disoproxil fumarate (TDF), lamivudine (3TC), emtricitabine (FTC), efavirenz (EFV)				
9. Functional newborn ambu bag and mask (sizes 0 and 1) within 2 meters of each delivery bed				
10. Oxygen for newborn use				
11. CPAP				
12. Functional autoclave				
13. Routine eye prophylaxis		☐ Normal storage [a]<br>☐ Protected from light [b]<br>☐ No expired drugs		
14. Vitamin K		☐ Normal storage [a]<br>☐ Protected from light [b]<br>☐ No expired drugs		
13. Hepatitis B vaccine		☐ 2°C -8°C<br>☐ No expired drugs		
14. Surgical gloves				

[a] Storage in dry, well-ventilated premises at temperatures of 15-25 C or, depending on climatic conditions, up to 30 C
[b] Oxytocin (unlike methergin) is not light sensitive but it is still good practice to protect it from light as there is a 7% loss in potency when exposed to light if stored at 21-25 C
[c] Recommended when the following conditions can be met: gestational age assessment can be accurately undertaken, preterm birth is considered imminent, there is no clinical evidence of maternal infection, adequate childbirth care is available, and the preterm newborn can receive adequate care if needed.
[d] Preterm prelabour rupture of the membranes is defined as rupture of the membranes before labour has begun in a pregnancy with a gestational age of less than 37 weeks

2.3 *Review activities of the EENC Team and hospital data in the previous 12 months*

Instructions

1. Obtain and review copies of EENC hospital documents for the previous 12 months, including notes or reports of EENC team meetings, quality improvement assessments and support visits. In **Checklist 5**, note if documentation supports regular EENC team meetings, EENC quality improvement reviews (based on needs found in the full EENC quality assessments and follow up).

2. Determine whether the EENC coaching database is being maintained and is up to date. If so, complete coaching coverage for main categories of worker and the total EENC coaching coverage for all staff.

3. Determine whether an EENC quality assessment has been conducted at least once in the previous 12 months. If so, obtain a copy of the findings.

4. Determine whether hospital impact data is being recorded.
    a. If yes, obtain a copy of the findings for the previous 12 months.

5. Provide feedback to EENC hospital team members on strengths and concerns. Discuss approaches to addressing problem areas and improving performance.

Checklist 6: Review activities of the EENC team and hospital data in the previous 12 months

1. In the previous 12 months:	
a. Has the hospital team been supported by hospital director or senior staff?	Y/N
b. Has the hospital team met regularly and documented meetings?	Y/N
c. Have two EENC quality assessments been conducted and documented?	Y/N
d. Has the EENC hospital plan been reviewed and updated at least quarterly?	Y/N
f. Facility has components of an EENC quality approach in place (Answer Y if b & c. = Y)	Y/N
2. Staff coaching database is available? If Yes,	Y/N
a. Number of delivery staff coached, n/N (%)	
b. Number of ward staff coached, n/N (%)	
c. Total staff coached, n/N (%)	
3. At least 1 full EENC hospital assessment from the previous 12 months is available? If Yes, obtain a copy.	Y/N
4. EENC Hospital impact database is available for the previous 12 months? If Yes, obtain a copy.	Y/N
5. How many maternal deaths occurred in the past 12 months?<br>5b. How many were reviewed?	
6. How many newborn deaths occurred in the past 12 months?<br>6b. How many were reviewed?	
7. How many stillbirths occurred in the last 12 months?<br>6b. How many were reviewed?	
8. Does the hospital have orders prohibiting promotion of infant formula and other linkages with milk formula companies?	Y/N
9. What percentage of staff has received education about responsibilities under the code of marketing of breastmilk substitutes? (%)	

2.4 Identify and Prioritize EENC Strengths and Areas for Improvement for EENC

Instructions

Call all the participants to a meeting including:

- Hospital Director
- head of OBGY ward
- Head of Paediatric Ward
- Delivery ward staff (doctor, midwife, nurse)
- NICU staff (doctor, nurse)_
- Hospital Administration
- Infection control

<u>Process of data analysis and planning actions</u>

G. Review action plan from the last monitoring (Use Table 2 from the last monitoring)
   1. Ask provincial / district staff to report progress (Record in the Table 2 “Status / Date”)
   2. Review repeated problems and reasons
   3. Ask the provincial / district team to discuss and agree on the actions to be taken with

timeframe and responsibilities (record in Table 2)

* Repeated problems have reasons of not implementing, so they cannot copy the same actions. They need to agree on realistic actions that they can act upon.

H. Review exit interview of mothers
I. Review chart reviews
J. Review direct observation
K. Review environmental hygiene (for external monitoring only)
L. Review medicines and supplies (for external monitoring only)

[Repeat the 1-5 following process for II to VI]

1. Ask provincial / district staff to report the result
2. Ask the provincial / district team to identify strengths (record in Table 1)
3. Ask the provincial / district team to discuss and agree on which areas for improvement to prioritize (record in Table 1)
4. Ask the provincial / district to discuss and agree on bottlenecks
5. Ask the provincial / district team to discuss and agree on actions to be taken with timeframe and responsibilities (record in Table 2)* Only if there are issues that cannot be solved within the facility, record in the column "Request for support"

Tips for the external facilitators:

4. Regulate the step-by-step process (Eg. If the provincial team jumped the discussion from selecting priority problems to solution, you need to bring them back)
5. Facilitate the discussion to be specific based on the data, and redirect it when they state bottleneck or solution that are general (We often observe general reasons and solutions that could come out without having specific data.)
6. Not tell them what are the problems, bottlenecks, solutions are: they should come from the team themselves

Table 1. Identifying and Prioritizing Strengths and Areas for Improvement for EENC

	Strengths	Areas for Improvement
Interviews		
Chart reviews		
Observation of delivery		
Observation of environmental hygiene		
Medicines and supplies		

Hospital Policy		

Table 2: Priority Actions for Improving EENC

Priority Actions	Person-responsible	Time	Status/ Date
Pending issues:<br>•<br>•			
Interviews:<br>•<br>•			
Chart reviews:<br>•<br>•			
Observation of delivery:<br>•<br>•			
Observation of environmental hygiene<br>•<br>•			

Medicines and supplies<br>•<br>•			
Hospital policies:<br>•<br>•			
**Request to Province/Central**<br>*** Most of the actions need to be taken by your own facility. This request is for an exceptional case only if you have crucial issues that cannot be solved in your facility.**			
