# Supplementary material for: Study protocol of a cluster randomized controlled trial to evaluate effectiveness of a system for maintaining high-quality early essential newborn care in Lao PDR
Source: BMC Health Serv Res. 2018 Jun 25;18:489. doi: 10.1186/s12913-018-3311-7 (PMC6019299; doi:10.1186/s12913-018-3311-7)
Supplement: Supplementary file 2 — Questionnaire on determinants to perform Early Essential Newborn Care. (DOCX 22 kb) [file 12913_2018_3311_MOESM2_ESM.docx]

Additional file 2：Questionnaire on determinants to perform Early Essential Newborn Care

Name of respondent _________________________

Date of fill in this questionnaire __________________________

District hospital________________________

Please answer each of the following questions by circling the number that best describes your opinion. Some of the questions may appear to be similar, but they do address somewhat different issues. Please read each question carefully.

1. For me to provide the EENC for every newborn I assist birth in this hospital on a regular basis is

harmful :___1__:___2__:___3__:___4__:___5__:___6__:___7__: beneficial

good :___1__:___2__:___3__:___4__:___5__:___6__:___7__: bad

pleasant :___1__:___2__:___3__:___4__:___5__:___6__:___7__: unpleasant

worthless :___1__:___2__:___3__:___4__:___5__:___6__:___7__: useful

1. My providing EENC for every newborn at this hospital on regular basis will result in preventing newborn morbidities and mortalities

Unlikely:___1__:___2__:___3__:___4__:___5__:___6__:___7__: Likely

1. For me, preventing newborn morbidities and mortalities is

Extremely undesirable:__1__:__2__:___3__:__4__:__5__:__6__:__7__: Extremely desirable

1. My providing EENC for every newborn at this hospital on regular basis will result in improving my clinical skills on birth assistance.

Unlikely:___1__:___2__:___3__:___4__:___5__:___6__:___7__: Likely

1. For me to keep up with my skills on birth assistance is

Extremely Extremely

undesirable:___1__:___2__:___3__:___4__:___5__:___6__:___7__: desirable

1. My providing EENC for every newborn at this hospital on regular basis will result in getting trust from patients.

Unlikely:___1__:___2__:___3__:___4__:___5__:___6__:___7__: Likely

1. For me, gaining trust from patients is

Extremely Extremely

undesirable:___1__:___2__:___3__:___4__:___5__:___6__:___7__: desirable

1. My providing EENC for every newborn at this hospital on regular basis will result in getting good evaluation.

Unlikely:___1__:___2__:___3__:___4__:___5__:___6__:___7__: Likely

1. For me, getting high rating in personal evaluation at the hospital is

Extremely Extremely

undesirable:___1__:___2__:___3__:___4__:___5__:___6__:___7__: desirable

1. Most people who are important to me think that

I should :___1__:___2__:___3__:___4__:___5__:___6__:___7__: I should not

provide the EENC for every newborn I assist birth in this hospital on a regular basis

1. It is expected of me that I provide the EENC for every newborn I assist birth in this hospital on a regular basis

strongly disagree :___1__:___2__:___3__:___4__:___5__:___6__:___7__: strongly agree

1. I feel under social pressure to provide the EENC for every newborn I assist birth in this hospital on a regular basis

strongly disagree :___1__:___2__:___3__:___4__:___5__:___6__:___7__: strongly agree

1. The director of this district hospital thinks that

I should not:___1__:___2__:___3__:___4__:___5__:___6__:___7__ : I should

provide the EENC for every newborn I assist birth in this hospital on a regular basis

1. Most of the health staff that assist childbirth in this hospital with whom I am acquainted provide the EENC to every newborn on a regular basis

definitely false:___1__:___2__:___3__:___4__:___5__:___6__:___7__: definitely true

1. My patients think that

I should not:___1__:___2__:___3__:___4__:___5__:___6__:___7__: I should

provide the EENC for every newborn I assist birth in this hospital on a regular basis

1. How much do you care what the director of this district health office/ hospital think you should do?

not at all :___1__:___2__:___3__:___4__:___5__:___6__:___7__: very much

1. How much do you care what your coworkers do?

not at all :___1__:___2__:___3__:___4__:___5__:___6__:___7__: very much

1. How much do you care what your patients think you should do?

not at all :___1__:___2__:___3__:___4__:___5__:___6__:___7__: very much

1. For me to provide the EENC for every newborn I assist birth in this hospital on a regular basis is

extremely easy :___1__:___2__:___3__:___4__:___5__:___6__:___7__: extremely difficult

1. I am confident that if I wanted to I could provide the EENC for every newborn I assist birth in this hospital on a regular basis.

definitely false :___1__:___2__:___3__:___4__:___5__:___6__:___7__: definitely true

1. Whether or not I provide the EENC for every newborn I assist birth in this hospital on a regular basis is completely up to me

strongly disagree:___1__:___2__:___3__:___4__:___5__:___6__:___7__: strongly agree

1. The decision to provide the EENC for every newborn in this hospital on a regular basis is beyond my control.

strongly disagree:___1__:___2__:___3__:___4__:___5__:___6__:___7__: strongly agree

1. How often do family obligations place unanticipated demands on your time working on EENC?

very rarely :___1__:___2__:___3__:___4__:___5__:___6__:___7__: very frequently

1. How often does work or employment place unanticipated demands on your time working on EENC?

very rarely :___1__:___2__:___3__:___4__:___5__:___6__:___7__: very frequently

1. How often is necessary equipment for EENC not available in this district hospital?

very rarely :___1__:___2__:___3__:___4__:___5__:___6__:___7__: very frequently

1. If I had family obligations that placed unanticipated demands on my time, it would make it

Much more much

difficult:___1__:___2__:___3__:___4__:___5__:___6__:___7__: easier

for me to provide EENC service for every newborn on a regular basis.

1. If work or employment placed unanticipated demands on my time, it would make it

much more much

difficult:___1__:___2__:___3__:___4__:___5__:___6__:___7__: easier

for me to provide EENC service for every newborn on a regular basis.

1. If necessary equipment for EENC is not available, I am

less likely :___1__:___2__:___3__:___4__:___5__:___6__:___7__: more likely

to provide EENC service for every newborn on a regular basis.

1. Given 10 pregnant women presenting for delivery, how many pregnant women would you expect to provide full EENC?

___0__:___1__:___2__:___3__:___4__:___5__:___6__:___7__:__8__:__9__:___10___

1. I plan to provide the EENC for every newborn I assist birth in this hospital on a regular basis

Strongly disagree :___1__:___2__:___3__:___4__:___5__:___6__:___7__: Strongly agree

1. I intend to provide full EENC for every newborn I assist birth in this hospital on a regular basis

Strongly disagree:__1__:___2__:___3__:___4__:___5__:___6__:___7__: Strongly agree

1. I will want to provide full EENC for every newborn I assist birth on a regular basis

Strongly disagree:__1__:___2__:___3__:___4__:___5__:___6__:___7__: Strongly agree

1. In the past three months, I provided full EENC for every newborn I assist birth on a regular basis

Strongly disagree:__1__:___2__:___3__:___4__:___5__:___6__:___7__: Strongly agree

1. During the past three months, what percentage of newborns you provided the EENC? During the past three months, I have provided the EENC for about ____ % of newborns I assisted birth in this hospital.
