# Supplementary material for: Study protocol of a cluster randomized controlled trial to evaluate effectiveness of a system for maintaining high-quality early essential newborn care in Lao PDR
Source: BMC Health Serv Res. 2018 Jun 25;18:489. doi: 10.1186/s12913-018-3311-7 (PMC6019299; doi:10.1186/s12913-018-3311-7)
Supplement: Supplementary file 3 — Written test on Early Essential Newborn Care. (PDF 1653 kb) [file 12913_2018_3311_MOESM3_ESM.pdf]

Additional file 3: Written test

Participant's name:.. District________________

Date:...

Choose the single best answer for each question

1. Which of the following is the correct statement for preparation prior to delivery?

    □ a. Ensure room temperature of 18℃

    □ b. Turn on fan and air-conditioning

    □ c. Open a window

    □ d. Place dry cloth on mother's abdomen

2. Delivering in the supine position during second stage of labour is best.

□ a. True

□ b. False

3. Applying fundal pressure (pushing down on the top of the uterus) is an effective means of supporting labour.

□ a. True

□ b. False

4. After a baby is born, you should call out the time of birth (accurate to minute and second), then what?

□ a. Clamp and cut the cord.

□ b. Thoroughly dry the baby.

□ c. Suction the baby's mouth and nose.

□ d. Hold the baby upside-down to let out the secretions.

5. What is correct statement for thorough drying

☐ a. Use a clean, dry cloth for wiping the baby

☐ b. Start wiping from arms and legs of the baby

☐ c. Wrap a baby with towels after drying to keep the baby warm.

☐ d. All of the above.

6. During thorough drying and stimulation of the baby, your rapid assessment shows she is crying. What is your next action?

☐ a. Suction the baby's mouth and nose.

☐ b. Clamp and cut the cord.

☐ c. Place the baby in skin-to-skin contact with the mother.

☐ d. Place the baby onto the breast.

7. For which reason(s) should the baby's mouth and nose be suctioned after thorough drying?

☐ a. The baby is breathing and the amniotic fluid is thickly stained with meconium and the baby is covered in meconium.

☐ b. The baby is not breathing and the amniotic fluid is thickly stained with meconium and the baby is covered in meconium.

☐ c. The baby is not breathing and there is no airway obstruction visible.

☐ d. All of the above.

8. What is the approximate capacity of a newborn's stomach? Circle A, B, C or D.

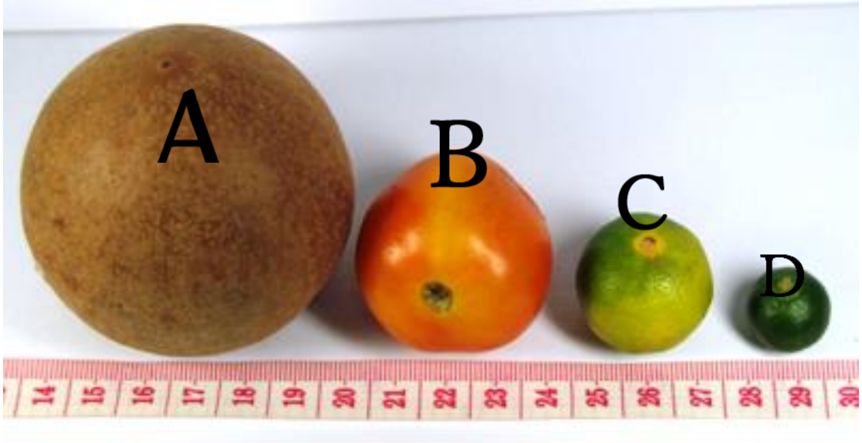

9. Which is the correct statements for skin- to-skin?

☐ a. Place the baby prone on the mother’s abdomen or chest.

☐ b. Cover the baby and mother with a clean warm cloth and head with a bonnet.

☐ c. Do not separate the baby from the mother as long as the baby is well.

☐ d. All of the above.

10. At least how long skin-to-skin should be continued after birth?

....

11. List 3 situations when skin-to-skin can be interrupted.

....

....

12. What is your next action after placing the baby in skin-to-skin contact with the mother when the baby is doing well?

☐ a. Examine the baby and provide eye care.

☐ b. Encourage breastfeeding.

☐ c. Bath the baby.

☐ d. Inject oxytocin after excluding a second baby and check cord pulsations

13. Which is the correct statements for cord clamping and cutting if the baby is breathing?

☐ a. Clamp the cord immediately after birth of the baby.

☐ b. Clamp the cord after cord pulsations have stopped.

☐ c. Do not change gloves before handling the cord if you are lone birth attendant.

☐ d. All of the above.

14. Choose the correct length between the first clamping site and the umbilical base.

☐ a. 1cm

☐ b. 2cm

☐ c. 5cm

☐ d. None of the above

15. A baby has feeding cues indicating she is ready to breastfeed immediately after birth.

☐ a. True

☐ b. False

16. List 3 signs a newborn baby is ready to breastfeed ("feeding cues").

....

....

17. List 3 signs a baby has good attachment to the breast.

....

....

18. List 3 things you should do to improve bag-and-mask ventilation.

....

....

19. When does a baby need bag-and-mask ventilation? After thorough drying for 30 seconds, the baby is:

☐ a. Not breathing.

☐ b. Having difficulty breathing (gasping respirations).

☐ c. Breathing but limp and very pale or blue in colour.

☐ d. All of the above.

20. A baby required bag-and-mask ventilation for 2 minutes. You have stopped bag-and-mask ventilation. He is now crying, breathing without difficulty, pink, and the heart rate is > 100 beats per minute.

What should you do now?

☐ a. Place the baby in direct skin-to-skin contact with the mother/do routine newborn care.

☐ b. Move the baby to an observational area and monitor breathing every 10 minutes.

☐ c. Give oxygen by nasal cannula or mask.

☐ d. Do all of the above.
