# Supplementary material for: Study protocol of a cluster randomized controlled trial to evaluate effectiveness of a system for maintaining high-quality early essential newborn care in Lao PDR
Source: BMC Health Serv Res. 2018 Jun 25;18:489. doi: 10.1186/s12913-018-3311-7 (PMC6019299; doi:10.1186/s12913-018-3311-7)
Supplement: Supplementary file 4 — Simulation test on Early Essential Newborn Care. (DOCX 24 kb) [file 12913_2018_3311_MOESM4_ESM.docx]

Additional file 4: Simulation test

**1) Delivery practice for the Breathing Baby**

Date: _______________ Location: _______________

Observation conducted by: _______________________________

Name of the monitored staff：＿＿＿＿＿＿＿＿＿＿＿＿＿＿＿＿＿　ID___________

| **Activity** | **Done** | **Partially done** | **Not done/Not satisfactory** |
| --- | --- | --- | --- |
| **Pre-Birth Preparation:** |  |  |  |
| 1. Checked room temperature; turned off fans and/or air conditioning |  |  |  |
| 1. Washed hands before touching any delivery area surfaces and handling equipment |  |  |  |
| 1. Placed dry cloth on abdomen |  |  |  |
| 1. Prepared the newborn resuscitation area |  |  |  |
| 1. Checked if newborn ambu bag and mask are functional |  |  |  |
| 1. Checked if a suction bulb is functional |  |  |  |
| 1. Washed hands before gloving for delivery |  |  |  |
| 1. Wore two pairs of sterile gloves (if same attendant handles the cord)^1^ |  |  |  |
| 1. Arranged forceps, cord clamp/ties in easy to use order |  |  |  |
| **Immediate Postpartum/Newborn Activities:** |  |  |  |
| 1. Called out time of birth (hours, minutes, seconds) |  |  |  |
| 1. Drying started within 5 sec after birth?   *Answer <5 sec (2 points), 5-10 sec (1 point), >10 sec (0 points) |  |  |  |
| 1. Dried the baby thoroughly (wiped the eyes, face, head, front, back, arms and legs) |  |  |  |
| 1. Removed the wet cloth |  |  |  |
| 1. Baby was in direct skin-to-skin contact |  |  |  |
| 1. Covered baby’s body with cloth and head with a hat |  |  |  |
| 1. Checked for a second baby |  |  |  |
| 1. Oxytocin IM given to mother within 1 minute |  |  |  |
| 1. 1^st^ pair of gloves removed^1^ |  |  |  |
| 1. Cord pulsations checked before clamping, clamped after cord pulsations stopped (usually 1 – 3 minutes) |  |  |  |
| 1. Clamp/tie placed at 2 cm, forceps at 5 cm from umbilical base |  |  |  |
| 1. Delivered placenta |  |  |  |
| 1. Counseled mother on feeding cues (drooling, mouth opening, tonguing/licking, rooting, biting hand, crawling, etc)   *Answer 1-2 mentioned (1 point), >2 mentioned (2 points) |  |  |  |
| **Total Score: (# Done x 2 + # Partial x 1)**  (maximum score possible = 44) |  |  |  |

^1^ If the mode of delivery is caesarean section or a separate birth attendant is available to handle the cord, indicate N/A here but enter or score the practice as a 'Yes' with two points even if only a single set of sterile gloves is worn by the health worker that delivers the baby. However, subtract 2 points if the gloves handling the cord were in any way not sterile.

**2) Delivery practice for the Non-Breathing Baby**

Date: _______________ Location: _______________

Observation conducted by: ______________________________

Name of the monitored staff：＿＿＿＿＿＿＿＿＿＿＿＿＿＿＿＿＿　ID___________

| **Activity** | **Done** | **Partially done** | **Not done/Not satisfactory** |
| --- | --- | --- | --- |
| **Pre-Birth Preparation:** |  |  |  |
| 1. Checked room temperature; turned off fans |  |  |  |
| 1. Washed hands before touching any delivery area surfaces and handling equipment |  |  |  |
| 1. Placed dry cloth placed on abdomen |  |  |  |
| 1. Prepared the newborn resuscitation area |  |  |  |
| 1. Checked if newborn ambu bag and mask are functional |  |  |  |
| 1. Washed hands before gloving for delivery |  |  |  |
| 1. Wore two pairs of sterile gloves (if same attendant handles the cord)^1^ |  |  |  |
| 1. Arranged forceps, cord clamp/ties in easy to use order |  |  |  |
| **Immediate Postpartum/Newborn Activities:** |  |  |  |
| 1. Called out time of birth (hours__, minutes__, seconds__) |  |  |  |
| 1. Drying started within 5 seconds after birth?   *Answer <5 sec (2 points), 5-10 sec (1 point), >10 sec |  |  |  |
| 1. Dried the baby thoroughly (wiped the eyes, mouth/nose, face, head, front, back, arms and legs) |  |  |  |
| 1. Removed the wet cloth |  |  |  |
| 1. Put baby in direct skin-to-skin contact |  |  |  |
| 1. Covered baby’s body with cloth and head with a hat |  |  |  |
| 1. Determined the baby was gasping or not breathing^1^ |  |  |  |
| 1. Called for help and informed the mother |  |  |  |
| 1. Removed first pair of gloves^1^ |  |  |  |
| 1. Quickly clamped and cut cord |  |  |  |
| 1. Moved baby to resuscitation area |  |  |  |
| 1. Covered baby quickly during after transfer |  |  |  |
| 1. Positioned head correctly to open airways |  |  |  |
| 1. Applied face mask firmly over chin, mouth & nose |  |  |  |
| 1. Gained chest rise within 1 minute of birth^2^:   Minutes___ : Seconds ___ |  |  |  |
| 1. Squeezed bag to give 30-50 breaths per min |  |  |  |
| 1. Maintained good chest rise throughout or took steps to improve ventilation |  |  |  |
| 1. After baby breathing well, stopped ventilation |  |  |  |
| 1. Returned to skin-to-skin contact, covered baby |  |  |  |
| 1. Checked for another baby |  |  |  |
| 1. Gave oxytocin IM to the mother |  |  |  |
| 1. Delivered placenta |  |  |  |
| 1. Counseled mother that baby is ok and on feeding cues |  |  |  |
| **Total Score: (# Done x 2 + # Partial x 1) [**maximum = 62]^3^ |  |  |  |

^1^ If the mode of delivery is caesarean section or a separate birth attendant is available to handle the cord, indicate N/A here but enter or score the practice as a 'Yes' with two points even if only a single set of sterile gloves is worn by the health worker that delivers the baby. However, subtract 2 points if the gloves handling the cord were in any way not sterile.

^2^ Only scored as 'Yes' or 'No', no partial.

^3^ Deduct 5 points if resuscitation is performed when it is not required: if the baby is not breathing but has muscle tone and a grimace, and then is not dried appropriately (either not immediately, not thoroughly or not at all)
