# Supplementary material for: Study protocol of a cluster randomized controlled trial to evaluate effectiveness of a system for maintaining high-quality early essential newborn care in Lao PDR
Source: BMC Health Serv Res. 2018 Jun 25;18:489. doi: 10.1186/s12913-018-3311-7 (PMC6019299; doi:10.1186/s12913-018-3311-7)
Supplement: Supplementary file 5 — Checklist of hospital environment facilitating implementation of Early Essential Newborn Care. (DOCX 55 kb) [file 12913_2018_3311_MOESM5_ESM.docx]

Additional file 5: Checklist of hospital environment

1. **Environmental Hygiene:** Delivery Room, Recovery Room, Neonatal Care Unit and Postnatal Care Room

| **Question** | **Delivery Room (s)** | | **Recovery room (s)** | **Neonatal Care Unit** | **PNC Room (s)** | **Comments** |
| --- | --- | --- | --- | --- | --- | --- |
| **Hand washing facilities and toilets for patients^1^:** |  | |  |  |  |  |
| 1. What is the total number of rooms? (N) |  | |  |  |  |  |
| 1. Rooms have at least one sink for washing hands available for use in each room?^2^ (n/N) |  | |  |  |  |  |
| 1. What is the total number of sinks? (N) |  | |  |  |  |  |
| 1. How many sinks: |  | |  |  |  |  |
| - 1. are clean? (n/N) |  | |  |  |  |  |
| - 1. have continuous clean, running water^3^? (n/N) |  | |  |  |  |  |
| - 1. have soap^4^ available? (n/N) |  | | | | | |
| - 1. have at least one method to dry hands: single-use towel, hand drier, reusable sterile towels |  | |  |  |  |  |
| 1. Rooms have adequate sink hand washing facilities available (n/N) (n= Y if each room has at least one sink and 4. a.-c. = 100% for all sinks in the room). |  | |  |  |  |  |
| 1. Rooms have at least one bottle of alcohol gel/hand rub available for use in the room? (n/N) |  | |  |  |  |  |
| 1. Rooms have adequate sink hand washing facilities AND alcohol hand gel available for use in the room (n/N). (n= Y if 5. = Y and 6. = Y). |  | |  |  |  |  |
| 1. How many toilets exist for patients? |  | |  |  |  |  |
| 8a. Are clean? |  | |  |  |  |  |
| 8b. Are functioning?  8c. Are not further than 30m from all users?  8d. Are not further than 5m from hand washing facility? |  | |  |  |  |  |
| **Newborn Resuscitation area:** | | | | | | |
| 1. How many delivery beds ? 2. How many delivery beds have a resuscitation area available within 2 m? |  | |  |  |  |  |
| 1. How many resuscitation areas are available? |  | |  |  |  |  |
| 1. How many resuscitation areas: |  | |  |  |  |  |
| 1. are clean and dry? |  | |  |  |  |  |
| 1. have newborn ambu bag and mask available? |  | |  |  |  |  |
| **Promotion of baby food company products** | | | | | | |
| 1. Are baby food company materials visible (posters, brochures, stickers, painted walls, etc) |  |  | |  |  |  |
| 1. Are there points of sale inside the grounds of the hospital? (minimart or direct staff selling) |  |  | |  |  |  |
| 1. Is there infant formula visible in the post-partum wards? |  |  | |  |  |  |
| 1. Have health workers been given any gifts from baby food companies (pens, notebooks, cups, pencil holders, meals etc) |  |  | |  |  |  |

^1^ To undertake a complete hand hygiene assessment, see 'Hand Hygiene Self-Assessment Framework' (WHO, 2010)

^2^ If more than one room is available in a category, report availability in each room separately. Note if alcohol gel/hand rub is available for staff use but not for use by patients and families.

^3^ A water supply that is either piped or from onsite storage, with appropriate disinfection, meeting appropriate safety standards for microbial and chemical contamination.

^4^ Soap: detergent-based products that contain no added antimicrobial agents or may contain these solely as preservatives. It may be in various forms including bar soap, tissue, leaf and liquid preparations.

1. **Availability of key medicines and supplies for EENC**

|  | **Available on the day of the review? Y or N** | **Stock condition?**  **No expired drugs? Equipment functional?** | **Stock records?**  **(Y or N)** | **# Stock-outs in last 12 months** |
| --- | --- | --- | --- | --- |
| - - - 1. Magnesium sulfate for severe pre-eclampsia and eclampsia, and fetal neuroprotection if gestational age <32 weeks |  | Normal storage ^a^  No expired drugs |  |  |
| - - - 1. Oxytocin for IM and parenteral use – immediately postpartum and for control of hemorrhage |  | 2°C -8°C  Protected from light ^b^  No expired drugs |  |  |
| - - - 1. Corticosteroids for women of 24 – 34 weeks of gestation at risk of preterm delivery ^c^ |  | Normal storage ^a^  Protected from light ^b^  No expired drugs |  |  |
| - - - 1. Injectable antibiotics for management of newborn sepsis |  | Normal storage ^a^  Protected from light ^b^  No expired drugs |  |  |
| - - - 1. Antibiotics for preterm pre-labour rupture of membranes^d^ |  | Normal storage ^a^  Protected from light ^b^  No expired drugs |  |  |
| - - - 1. Syphilis test kits |  |  |  |  |
| - - - 1. HIV test kits |  |  |  |  |
| - - - 1. First line ART regimen: tenofovir disoproxil fumarate (TDF), lamivudine (3TC), emtricitabine (FTC), efavirenz (EFV) |  |  |  |  |
| - - - 1. Functional newborn ambu bag and mask (sizes 0 and 1) within 2 meters of each delivery bed |  |  |  |  |
| - - - 1. Oxygen for newborn use |  |  |  |  |
| - - - 1. CPAP |  |  |  |  |
| - - - 1. Functional autoclave |  |  |  |  |
| - - - 1. Routine eye prophylaxis |  | Normal storage ^a^  Protected from light ^b^  No expired drugs |  |  |
| - - - 1. Vitamin K |  | Normal storage ^a^  Protected from light ^b^  No expired drugs |  |  |
| 13. Hepatitis B vaccine |  | 2°C -8°C  No expired drugs |  |  |
| 1. Surgical gloves |  |  |  |  |

^a^ Storage in dry, well-ventilated premises at temperatures of 15-25 C or, depending on climatic conditions, up to 30 C

^b^ Oxytocin (unlike methergin) is not light sensitive but it is still good practice to protect it from light as there is a 7% loss in potency when exposed to light if stored at 21-25 C

^c^ Recommended when the following conditions can be met: gestational age assessment can be accurately undertaken, preterm birth is considered imminent, there is no clinical evidence of maternal infection, adequate childbirth care is available, and the preterm newborn can receive adequate care if needed.

^d^ Preterm prelabour rupture of the membranes is defined as rupture of the membranes before labour has begun in a pregnancy with a gestational age of less than 37 weeks

1. **Hospital’s level of commitment and activities of the EENC team**

| 1. In the previous 12 months: |  |
| --- | --- |
| a. Has the hospital team been supported by hospital director or senior staff? | Y/N |
| b. Has the hospital team met regularly and documented meetings? | Y/N |
| c. Have two EENC quality assessments been conducted and documented? | Y/N |
| d. Has the EENC hospital plan been reviewed and updated at least quarterly? | Y/N |
| - 1. Facility has components of an EENC quality approach in place (Answer Y if b & c. = Y) | Y/N |
| 1. Staff coaching database is available? If Yes, | Y/N |
| - 1. Number of delivery staff coached, n/N (%) |  |
| - 1. Number of ward staff coached, n/N (%) |  |
| - 1. Total staff coached, n/N (%) |  |
| 1. At least 1 full EENC hospital assessment from the previous 12 months is available? If Yes, obtain a copy. | Y/N |
| 1. EENC Hospital impact database is available for the previous 12 months? If Yes, obtain a copy. | Y/N |
| 1. How many maternal deaths occurred in the past 12 months?   5b. How many were reviewed? |  |
| 1. How many newborn deaths occurred in the past 12 months?   6b. How many were reviewed? |  |
| 1. How many stillbirths occurred in the last 12 months?   6b. How many were reviewed? |  |
| 1. Does the hospital have orders prohibiting promotion of infant formula and other linkages with milk formula companies? | Y/N |
| 1. What percentage of staff has received education about responsibilities under the code of marketing of breastmilk substitutes? (%) |  |
