# Supplementary material for: Study protocol of a cluster randomized controlled trial to evaluate effectiveness of a system for maintaining high-quality early essential newborn care in Lao PDR
Source: BMC Health Serv Res. 2018 Jun 25;18:489. doi: 10.1186/s12913-018-3311-7 (PMC6019299; doi:10.1186/s12913-018-3311-7)
Supplement: Supplementary file 6 — General Information Questionnaire. (DOCX 20 kb) [file 12913_2018_3311_MOESM6_ESM.docx]

Additional file 6：General Information Questionnaire

Date ___________________

Name of respondent_____________________

District_________________, Province____________________

1. When is your birthday?

Date of birth ______/_________/________(DD/MM/YY)

Age______

1. Sex Male /Female
2. Marital status　　　Married/Single/Divorced
3. How many children do you have in your family? ___________
4. What is your ethnicity? _________________
5. What is your title?

□1. Specialized doctor (specify which area _________________)

□2. General doctor

□3. Midwife

□4. Nurse

□ 5. Assistant nurse

□ 6. Medical assistant

□ 7. Others (Specify ____________________)

1. What is your current position in the hospital? ______________

When did you move to the current hospital? Year _______Month________

And how many years have you worked for supporting childbirth? (regardless of your working place) ____________year
