# Supplementary material for: Study protocol of a cluster randomized controlled trial to evaluate effectiveness of a system for maintaining high-quality early essential newborn care in Lao PDR
Source: BMC Health Serv Res. 2018 Jun 25;18:489. doi: 10.1186/s12913-018-3311-7 (PMC6019299; doi:10.1186/s12913-018-3311-7)
Supplement: Supplementary file 7 — Informed consent form. (DOCX 23 kb) [file 12913_2018_3311_MOESM7_ESM.docx]

Additional file 7: Informed consent form

Consent form

To Dean of Teikyo University Graduate School of Public Health

Title of the study：Building the system for maintaining high quality of Early Essential Newborn Care in the Western Pacific Region

I have received the explanation about the following items with a written document and agreed to participate in this research with my free will by fully understanding the explanation.

Items explained:

- Research objectives and its meaning
- Research targets and methods
- Freedom of participation and cancellation
- Responsible researcher and research organization
- Place and Duration of the research
- How to deal with data in the research
- How to deal with research results
- Financial source of the research
- Conflict of interest
- The burden and incentives of research participants
- Potential profit, disadvantage, adverse event that might happen to research participants and correspondence
- Conditions for cancellation of research
- Contact information of researchers

Date / /

Name （self-written）

　　　　　　　　　　　　　　　　　　　　　　　　　　　　　　　　　　　　　signature

Department of the explainer

Position/name of the explainer（self-written）　　　　　　　　　signature

Consent withdrawal form

To Dean of Teikyo University Graduate School of Public Health

Title of the study：Building the system for maintaining high quality of Early Essential Newborn Care in the Western Pacific Region

I gave my consent to participate in this research after receiving explanation, but I would like to withdraw it.

Date / /

Name （self-written）

　　　　　　　　　　　　　　　　　　　　　　　　　　　　　　　　　　　　　signature

Regarding the handling of research information, I request as follows.

□ I will allow you to use information I provided until the withdrawal of consent for research.

□I would like you to discard all information I provided until my consent withdrawal

Contact for any inquiries about this research

　Contact person in Lao PDR：Ms. Outhevanh KOUNNAVONGSA

Technical officer
Mother and Child Health Care Unit

World Health Organization, Vientiane capital, Lao PDR

Tel: +856-21-353902-4 Ext 81856

　Contact person in Japan：Dr. Sayaka HORIUCHI, Assistant professor

Dr. Kenzo TAKAHASHI, Associate professor

Teikyo University Graduate School of Public Health

Address：2-11-1 kaga, Itabashi, Tokyo, Japan

TEL：+81 3-3964-1211 Ext 46215
